# Tri-specific molecularly imprinted lysosomal nanodegrader enables synergistic therapy of cytokine storm

**DOI:** 10.1039/d5sc04757a

**Published:** 2025-09-23

**Authors:** Jingran Chen, Weihua Lu, Ying Li, Zhanchen Guo, Qian Liu, Weiwei Liu, Lisheng Wang, Zhen Liu

**Affiliations:** a State Key Laboratory of Analytical Chemistry for Life Science, School of Chemistry and Chemical Engineering, Nanjing University 163 Xianlin Avenue Nanjing 210023 China zhenliu@nju.edu.cn; b Department of Biochemistry, Microbiology and Immunology, Faculty of Medicine, University of Ottawa Ottawa Ontario K1H 8M5 Canada

## Abstract

The excessive release of proinflammatory cytokines, especially IL-6 and TNF-α, plays a critical role in the pathogenesis of cytokine release syndrome, also known as a cytokine storm. Although targeted cytokine degradation holds great promise in controlling the overwhelming inflammation, its development for early intervention in cytokine storm is impeded by several challenges, including limited modularity, high preparation cost, complex synthetic procedures, and a limited number of substrates amenable to degradation. Herein, we developed a tri-specific molecularly imprinted polymer (tsMIP)-based lysosome-targeted chimera for simultaneous targeted degradation of IL-6 and TNF-α towards synergistic anti-inflammatory therapy of cytokine storm. The tsMIP was synthesized using N- or C-terminal nonapeptide epitopes of IGF2R, IL-6 and TNF-α as the templates, enabling high-affinity binding to all three proteins. Following rational administration during cytokine storm, tsMIP first captured the cytokines IL-6 and TNF-α, then targeted IGF2R on the cell surface, and finally transported the cytokines into lysosomes for degradation. This treatment significantly reduced the levels of the pro-inflammatory cytokines and the downstream phosphorylated STAT3/NF-κB p65 proteins as well, indicating effective lysosomal degradation and downstream signaling pathway blockade. *In vivo* verification in an acute lung injury mouse model, as a typical example of cytokine releasing syndrome, demonstrated potent inflammation mitigation of intranasal tsMIP administration. As a proof-of-concept, this study highlights the potential of a tri-specific molecularly imprinted lysosomal nanodegrader in achieving synergistic anti-inflammation effects, offering a versatile strategy for treating cytokine storm and other infectious and inflammatory diseases.

## Abstract 2

A tri-specific molecularly imprinted polymer-based lysosome-targeted chimera was developed for synergistic anti-inflammatory therapy, effectively reducing the level of IL-6, TNF-α, and downstream p-STAT3/NF-κB p65, thereby alleviating cytokine storm.

## Graphical abstract

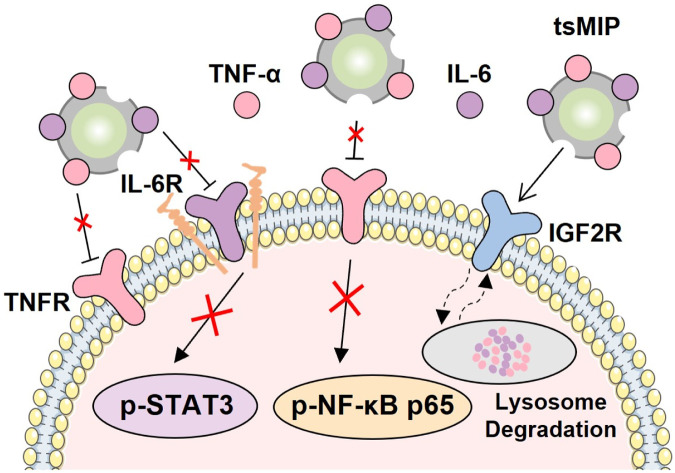

## Introduction

The cytokine release syndrome (CRS), originating from a “cytokine storm”, is a systemic inflammatory response caused by excessive or uncontrolled release of pro-inflammatory cytokines, which causes severe damage to the immune system.^1^ The typical example is the severe acute respiratory distress syndrome (ARDS) or acute lung injury (ALI), which is a lethal syndrome caused by pneumonia or sepsis and is also the major phenotype following respiratory viral infections, including SARS-COV, MERS, SARS-CoV-2, and many others.^2,3^ In such cases, the respiratory system becomes severely compromised, leading to alveolar edema, hypoxemia, dyspnea, and a systemic inflammatory response syndrome, eventually causing immune system dysfunction.^4^ The severe consequences caused by cytokine storms are profound and cannot be underestimated. However, the complexity of cytokine interactions and the multiplicity of cytokine targets make alleviating these storms particularly challenging.^5,6^ Therefore, it is imperative to elucidate the intricate network driving cytokine storm and to precisely target the key cytokines in order to develop effective treatment for CRS-related diseases.

In the onset and progression of cytokine storms, interleukin-6 (IL-6) and tumor necrosis factor (TNF-α) play pivotal roles. IL-6 is a key mediator of acute inflammatory responses and is involved in various immunopathological diseases.^7^ In CRS, IL-6 consistently increases as immune dysregulation progresses, making it a critical indicator for both the severity and prognosis of cytokine storms, with the IL-6–STAT3 axis being the primary cytokine pathway involved.^8^ TNF-α, a central cytokine in inflammatory reactions, can directly induce the expression of inflammatory genes or indirectly promote cell death, further driving inflammatory immune responses and disease progression.^9^ As a potent multifunctional proinflammatory molecule, TNF-α induces the expression of NF-κB, stimulates other cytokines such as IL-6, and enhances systemic inflammation.^10^ Together, IL-6 and TNF-α take the lead in CRS and have a synergistic effect, making them promising therapeutic targets for the treatment of cytokine storms.

Currently, the main treatment strategy for cytokine storms relies on monoclonal antibodies (MAbs) and small molecule medicines, both of which can reduce proinflammatory cytokine signaling and improve clinical outcomes. Cytokine-specific Mab therapies have gained wide acceptance due to their high specificity and efficiency, such as siltuximab targeting IL-6, tocilizumab targeting the IL-6 receptor (IL-6R) and infliximab targeting TNF-α.^11^ However, the usage of MAbs encounters certain risks, including acute anaphylaxis, serum disorders and antibody resistance.^12,13^ Additionally, high cost, stringent storage and transportation requirements limit their broader application.^14^ Small molecule drugs, such as chloroquine, colchicine and corticosteroids, offer alternatives^15,16^ but require high doses due to their non-selective properties, increasing the risk of systemic multi-organ damage.^17^ Besides, while the current therapies for CRS, such as tocilizumab and small molecules including corticosteroids mentioned above, are effective in many clinical cases, they have significant limitations such as limited cytokine targeting, delayed treatment initiation, potential for immunosuppression, rebound of symptoms, and lack of alternatives for refractory cases. Continued research into broader, multi-cytokine targeting approaches and strategies that preserve the efficacy of immunotherapy while controlling CRS is crucial. Therefore, new technologies that could overcome the disadvantages of current therapies and effectively suppress excessive multiple cytokine activity are urgently needed.

The COVID-19 pandemic has promoted the anti-inflammatory related research, with nanotechnology playing an increasingly important role.^18^ Utilizing biomaterial–antibody complexes or cell membrane-derived nanoparticles, overexpressed proinflammatory cytokines can be efficiently neutralized.^19^ However, the high cost and complex preparation process hinder their clinical application. The recent development of lysosome-targeted chimeras (LYTACs) has enabled the targeted degradation of extracellular proteins,^20–24^ suggesting that the degrading pro-inflammatory cytokines could be a promising strategy to treat CRS.^25^ As an appealing antibody mimic, molecularly imprinted polymers (MIPs) exhibit several advantages, including lower cost, easier storage, simpler preparation and multi-target binding capability compared to MAbs.^26–29^ Introducing MIPs as target binders into LYTACs not only provides antibody-like high affinity but also simplifies the construction of LYTACs, serving as a brand-new modular design platform for any substrate of interest, making the simultaneous degradation of secretory proteins and membrane proteins possible as well. Moreover, the easy internalization and multi-target binding capability of MIPs^30–32^ may result in a better degradation efficacy than traditional LYTACs.

**Scheme 1:**
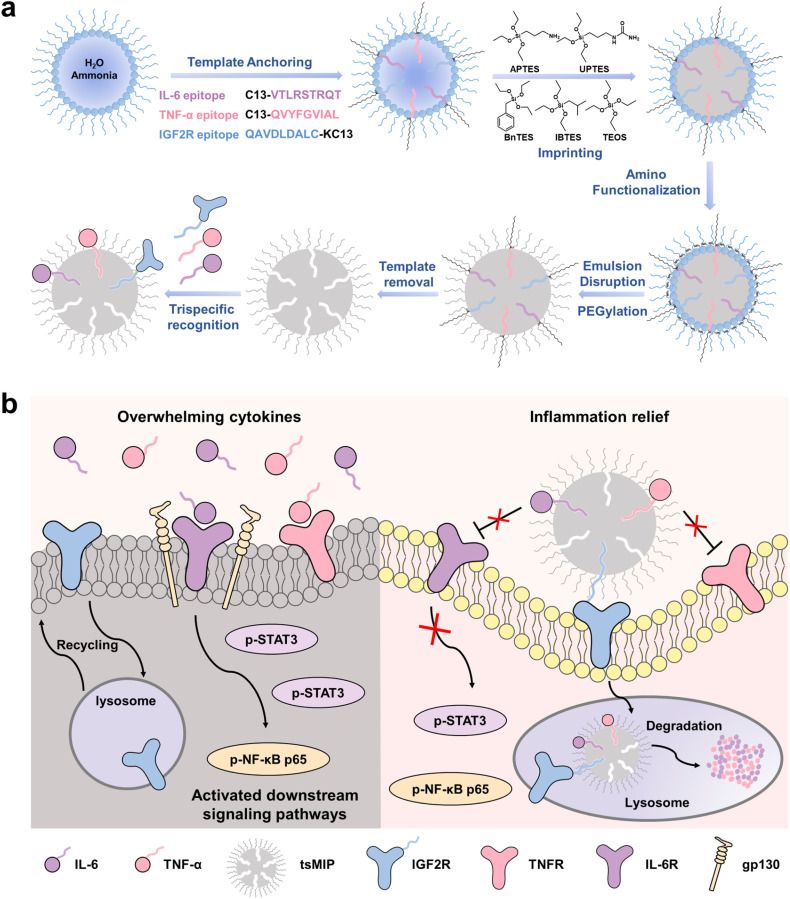
Schematic illustration of tsMIP-induced simultaneous degradation of extracellular proinflammatory cytokines IL-6 and TNF-α for synergistic therapy of cytokine storm. (a) Schematic of the synthesis route of tsMIP. (b) Mechanism of action of tsMIP in cytokine storm.

In light of the development of trispecific antibodies recognizing multiple targets in recent years,^33–35^ we designed an unprecedented trispecific MIP (tsMIP) to achieve synergistic degradation of proinflammatory cytokines, specifically IL-6 and TNF-α. The tsMIP was rationally designed and engineered using multi-template epitope imprinting to bind IL-6, TNF-α and the lysosomal targeting receptor IGF2R. The principle is illustrated in Scheme 1. Upon binding to IGF2R, the cytokines bound to tsMIP are internalized by the cells and transported to lysosomes for degradation. Hence, the expression of target cytokines, including IL-6 and TNF-α, along with downstream phosphorylated STAT3 and NF-κB p65 proteins, is significantly decreased. An acute lung injury (ALI) Balb/c mouse model was employed as an example for CRS, because ALI/ARDS is a typical example of immune dysregulation marked by an excessive inflammatory response, and due to its complex etiology, rapid progression, and high mortality, effective treatment options for ALI/ARDS are extremely limited.^36,37^*In vivo* verification demonstrated the potent synergistic anti-inflammatory effect of the tsMIP. This tsMIP-based LYTAC is the first reported antibody-free platform that enables simultaneous targeted degradation of two critical cytokines in CRS using nanomaterials. This innovative LYTAC platform offers synergistic anti-inflammatory effects and could be extended to other cytokines and chemokines, opening new avenues for treating infectious and inflammatory diseases.

## Results and discussion

### Bioinformatics analysis

**Fig. 1:**
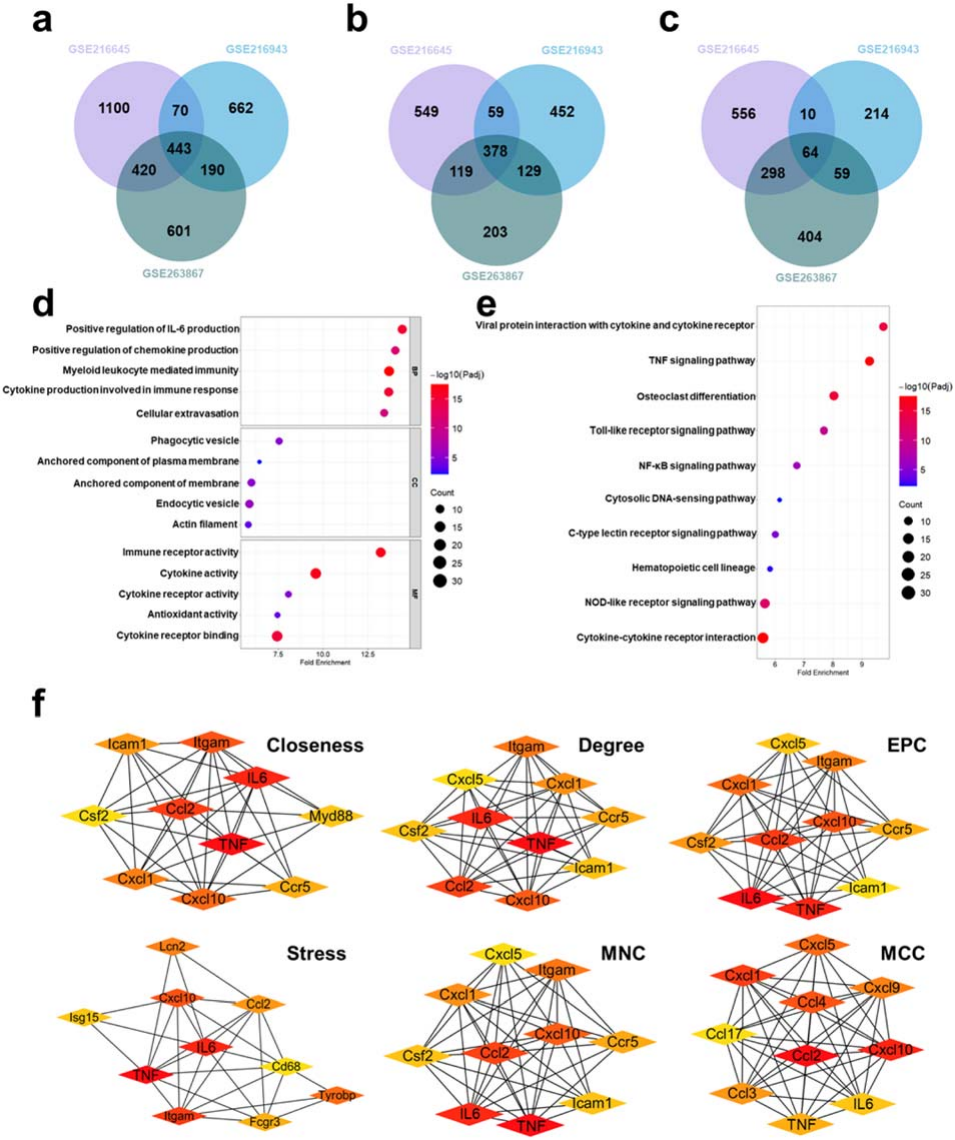
Bioinformatics analysis unveils critical cytokine secretion in acute lung injury. (a) Differentially expressed genes identified from the three datasets. (b) The significantly upregulated genes across the three databases. (c) The significantly downregulated genes across the three databases. (d) GO analysis (biological process, cellular component, and molecular function) of differentially expressed genes. (e) KEGG enrichment analysis of differentially expressed genes. (f) Hub genes analysis using various algorithms.

To clarify the critical cytokines in the intricate cytokine storm network, especially within the acute lung injury disease model, bioinformatics analysis was conducted. RNA-Seq datasets (GSE216645, GSE216943 and GSE263867) were downloaded from the Gene Expression Omnibus (GEO), and then differentially expressed genes (DEGs) were identified using the R programming language. Among the 443 overlapping genes, 378 genes were found significantly upregulated, while 64 downregulated (Fig. 1a–c and S1). These DEGs were further analyzed using the Gene Ontology (GO) and Kyoto Encyclopedia of Genes and Genomes (KEGG) databases, which revealed the gene interaction and corresponding signal pathways. As shown in Fig. 1d and e, cytokines such as IL-6 and TNF families, chemokines, and receptors including immune- and cytokine-related receptors, as well as pathways such as NF-κB and TNF are significantly upregulated. A comprehensive protein–protein interaction (PPI) network was then constructed, with blue nodes representing downregulated genes and red nodes representing upregulated genes (Fig. S2a). Hub genes within the PPI network were identified using different algorithms (Fig. 1f), including closeness, degree, stress, edge percolated component (EPC), maximum neighborhood component (MNC), and maximal clique centrality (MCC), with their upregulated intersections displayed in a Venn diagram. As shown in Fig. S2b, cytokines including IL-6 and TNF families, as well as chemokines including CCL2 and CXCL10, are upregulated. Given the synergistic effects of IL-6 and TNF-α,^10^ and their involvement in downstream signaling pathways, IL-6 and TNF-α hold a central role in CRS and warrant further investigation.

### Preparation and characterization of tsMIP

**Fig. 2:**
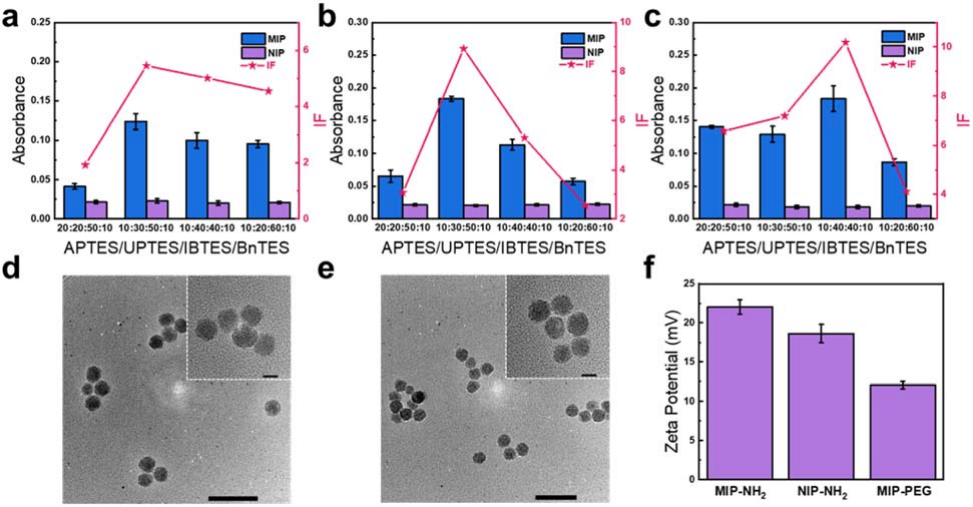
Synthesis and characterization of IL-6, TNF-α and IGF2R terminal epitope-imprinted tsMIP. (a–c) Optimization of functional monomer ratio for individual epitopes: (a) IGF2R epitope, (b) TNF-α epitope, and (c) IL-6 epitope. (d and e) TEM images of PEG functionalized tsMIP (d) and NIP (e). The enlarged images of the nanoparticles are shown within the dotted box. (f) Zeta potentials of MIP-NH_2_, NIP-NH_2_, and MIP-PEG. Error bars represent standard deviations (*n* = 3).

The tsMIP was prepared according to our recent approach called reverse microemulsion-confined epitope-oriented surface imprinting and cladding (ROSIC) with appropriate modifications.^38^ The synthesis procedure is illustrated in Scheme 1a. Since TNF-α exists in both soluble form (sTNF-α) located outside the cell and transmembrane form (tmTNF-α),^39^ the C-terminal nonapeptide (QVYFGVIAL) of the extracellular region of TNF-α was selected as the template to enable degradation of both forms simultaneously. For IL-6, a secretory protein,^40^ the C-terminal nonapeptide (VTLRSTRQT) was selected as the template. To target IGF2R, a transmembrane protein,^41^ the N-terminal nonapeptide (QAVDLDALC) of its extracellular region was used as the template. The interactions among the three epitopes were first evaluated through molecular docking analysis. The results revealed the presence of π–alkyl hydrophobic interactions and electrostatic forces (Fig. S3), with a calculated binding free energy of −3.133 kcal mol^−1^. This relatively weak binding affinity (binding free energies less negative than −4 kcal mol^−1^ are generally indicative of very weak interactions) suggests that the peptides are unlikely to form stable complexes and are more likely to remain unbound in solution. As supporting evidence, circular dichroism (CD) analysis was performed. As shown in Fig. S4, the CD spectrum of the mixed peptide solution exhibited peak positions identical to those of the individual peptide solutions, showing a random coil at around 200 nm, without the appearance of any new characteristic peaks. Moreover, the experimentally observed mixed spectrum was approximately equal to the theoretical sum of the individual spectra, indicating that the three peptides maintained their distinct free conformations in solution and did not form stable polymeric clusters. Then, appropriate ratios of functional monomers were carefully selected according to the properties and constitution of these peptides, with detailed structures shown in Fig. S5 and S6. To enhance biosafety, stability and prolong *in vivo* circulation, the tsMIP was modified with polyethylene glycol (PEG). Several tsMIPs with different functional monomer ratios were synthesized. And the corresponding imprinting effect was evaluated using the imprinting factor (IF) as an indicator, calculated as the ratio of the amount of the epitope captured by tsMIP and non-imprinted nanoparticles (NIP) for each peptide. Four functional monomer ratios (APTES/UPTES/IBTES/BnTES) were optimized. As shown in Fig. 2a–c, the 10 : 30 : 50 : 10 ratio demonstrated the strongest binding to IGF2R and TNF-α and the second-best to IL-6. To maximize simultaneous binding to all three targets, the compromise monomer ratio 10 : 30 : 50 : 10 was selected for subsequent experiments, under which the IF values were found to be 5.5, 8.9 and 7.2 for the IGF2R epitope, the TNF-α epitope, and the IL-6 epitope, respectively, which are well acceptable.

Transmission electron microscopy (TEM) was used to characterize the morphology of the tri-template imprinted MIP (tsMIP), bi-template imprinted MIP (MIP_IL-6&TNF-α_), single template imprinted MIP (MIP_IGF2R_) and NIP. As shown in Fig. 2d, e and S7, after PEG modification, the nanoparticles exhibited improved monodispersity and a well-defined spherical shape with a mean diameter of about 30 nm (Fig. S8). Zeta potential analysis revealed that amino-functionalized nanomaterials carried a positive charge, with MIP-NH_2_ exhibiting higher zeta potential than NIP-NH_2_ due to more exposed surface cavities. After PEG modification, the zeta potential of MIP-PEG was reduced, as some surface charges were shielded (Fig. 2f). Additionally, the PEG modification retained nanoparticles stability and dispersibility in the initial 3 days (Fig. S9).

Cell viability assays on RAW264.7 cells confirmed the biocompatibility of the nanoparticles. As shown in Fig. S10, when the concentration of tsMIP/NIP increased from 12.5 to 400 μg mL^−1^, the cell survival rate still remained above 80%, demonstrating good biocompatibility. Through rational template selection, monomer ratio optimization and PEG modification, the tsMIP demonstrated strong binding ability and biosafety, laying a solid foundation for its effective and safe application *in vivo*.

### Cytokine-capturing capability of tsMIP

**Fig. 3:**
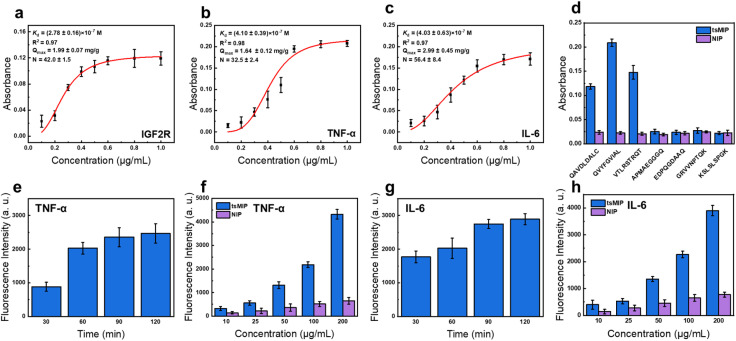
Characterization of tsMIP binding properties. (a–c) Isothermal binding curve of tsMIP towards (a) the N-terminal epitope of IGF2R, (b) the C-terminal epitope of TNF-α, and (c) the C-terminal epitope of IL-6. (d) The selectivity of tsMIP toward different peptides. (e and f) Dependence of the fluorescence intensity of TNF-α captured by tsMIP from cell culture medium on the incubation time (e) and the concentration of tsMIP (f). (g and h) Dependence of the fluorescence intensity of IL-6 captured by tsMIP from cell culture medium on the incubation time (g) and the concentration of tsMIP (h). Error bars represent standard deviations (*n* = 3).

After optimizing the tsMIP monomer ratio, the binding affinity towards the three target epitopes was evaluated by plotting and fitting isothermal adsorption curves. As shown in Fig. 3a–c, tsMIP showed high binding affinity to all three epitopes with *K*_d_ values in the range of 10^−7^ M level, saturated adsorption capacities (*Q*_max_) of approximately 2 mg g^−1^, and 30–60 binding sites per tsMIP nanoparticle for each epitope. The *Q*_max_ and binding sites of tsMIP were estimated according to the standard curves (Fig. S11) and isothermal adsorption curves (Fig. 3a–c, Hill equation) of the epitopes. Detailed calculations are described in the SI. Additionally, tsMIP exhibited excellent selectivity for the target epitopes, with minimal binding to non-targeting epitopes (Fig. 3d).

With satisfied binding affinity and selectivity established, the corresponding cytokine-capturing capability of tsMIP was then investigated. To effectively induce the degradation of extracellular IL-6 and TNF-α, tsMIP must first capture sufficient amounts of these cytokines and simultaneously facilitate their internalization into the cells for lysosome degradation. As a proof of concept, RAW264.7 cells (a kind of murine-derived macrophage) were stimulated with lipopolysaccharide (LPS) to generate large amounts of cytokines. The cell culture medium was collected and incubated with tsMIP solution to capture cytokines, which were then labeled with the fluorescent antibody for visualization. As shown in Fig. 3e and f, the fluorescence intensity steadily increased with incubation time, reaching saturation at around 2 h.

Next, varying concentrations of tsMIP were incubated with the cytokine-rich cell culture medium for 2 h. As the concentration of tsMIP increased, the amount of TNF-α captured also increased significantly compared to NIP, with a similar trend observed for IL-6 (Fig. 3g and h). Collectively, these results demonstrate that tsMIP exhibits strong cytokine-capturing capability, highlighting its potential for targeted cytokine degradation.

### Cell uptake and lysosome colocalization analysis

**Fig. 4:**
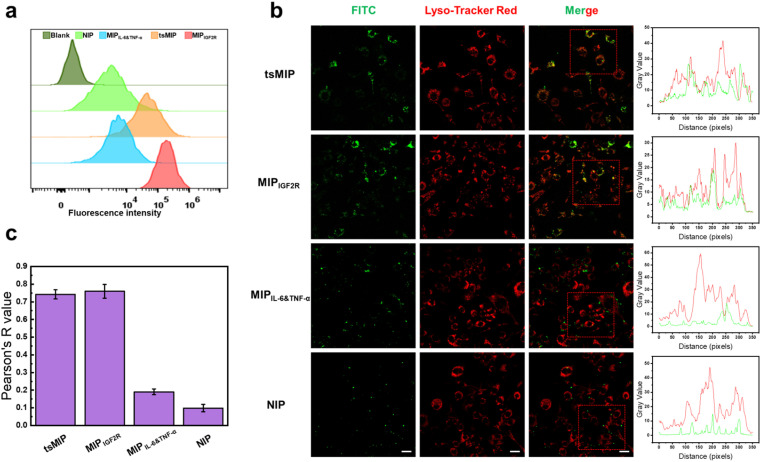
Analyses of cell uptake and lysosomal colocalization. (a) Cellular uptake of tsMIP and control NPs analyzed by flow cytometry. (b) Confocal fluorescent microscope images and corresponding lysosomal co-localization analysis for tsMIP, MIP_IGF2R_, MIP_IL-6&TNF-α_ and NIP. Scale bars = 20 μm. (c) Statistics of co-localization values between lysosomes and tsMIP/control NPs. Error bars represent standard deviations (*n* = 3).

A key requirement for tsMIP to achieve intracellular degradation of TNF-α and IL-6 is the efficient cell uptake and lysosome colocalization. To assess this, the uptake of various nanoparticles, including MIP_IGF2R_, MIP_IL-6&TNF-α_, tsMIP and NIP, by RAW 264.7 cells was examined. As shown in Fig. 4a and S12, NIP exhibited the lowest cell uptake among the four kinds of nanoparticles; MIP_IGF2R_ exhibited the highest uptake, which is attributed to its ability to bind IGF2R; tsMIP exhibited the second highest uptake, which is attributed to its ability to bind IGF2R but its binding capacity to IGF2R was reduced to some extent due to the occupancy of its surface by the binding cavities for the other two templates; MIP_IL-6&TNF-α_ showed a slightly higher uptake than NIP, likely due to the expression of tm TNF-α on the cell membrane, which facilitates binding to cells and enhances its phagocytosis. Furthermore, the lysosome co-localization of different nanoparticles was investigated. As shown in Fig. 4b and c, tsMIP and MIP_IGF2R_ exhibited the highest lysosome colocalization, reflected by strong Pearson's *R* values, attributed to the presence of IGF2R binding cavities. Conversely, MIP_IL-6&TNF-α_ and NIP nanoparticles exhibited poor lysosome colocalization due to the lack of IGF2R-specific binding cavities. The high lysosome retention of tsMIP lays a solid foundation for the subsequent lysosome degradation of extracellular cytokines.

### Targeted degradation of cytokines and downstream signal pathway analysis

TNF-α exists in both a transmembrane form (tmTNF-α) and a soluble form (sTNF-α). The degradation of each form was investigated separately. First, the targeted degradation of tmTNF-α was evaluated by flow cytometry and confocal fluorescence microscopy. RAW264.7 cells were stimulated with LPS and then incubated with increasing concentrations of tsMIP for 12 h before tmTNF-α immunostaining. As shown in Fig. S13a and b, the degradation efficiency of tmTNF-α increased in a dose-dependent manner, reaching 68% at a tsMIP concentration of 200 μg mL^−1^. Additionally, RAW264.7 cells were treated with various control nanoparticles (200 μg mL^−1^) for 12 h (Fig. S13c and d). Among the groups, tsMIP showed the greatest degradation efficiency (69%). As a comparison, the MIP_IL-6&TNF-α_ treatment group exhibited a tmTNF-α degradation efficiency of approximately 25%, which is likely due to the presence of TNF-α binding cavities on the nanoparticles. Binding of MIP_IL-6&TNF-α_ to cells may enhance the cellular uptake of nanoparticles, thereby decreasing tmTNF-α expression to some extent. Confocal fluorescence microscope results (Fig. S13e) also confirmed this finding, with the tsMIP treatment group showing the most significant tmTNF-α degradation, while the MIP_IL-6&TNF-α_ treatment group also showed some tmTNF-α degradation compared to the NIP and untreated groups.

**Fig. 5:**
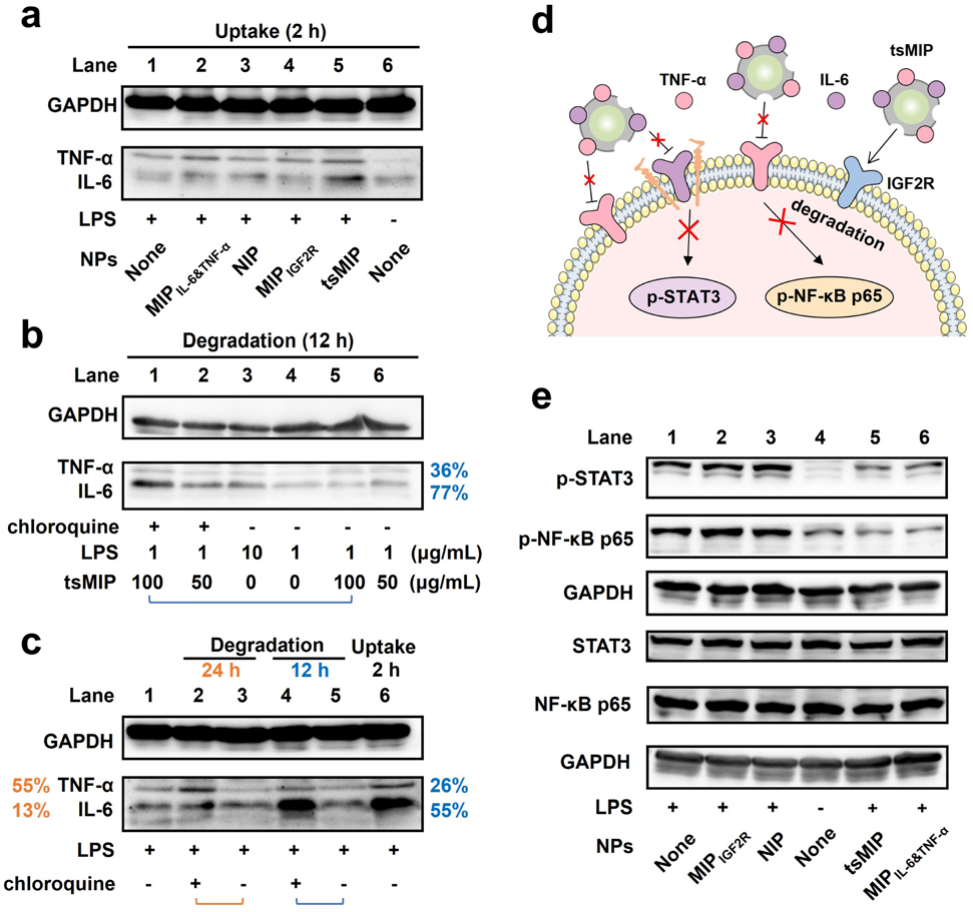
Western blot validation of degradation analysis. (a) The expression of TNF-α and IL-6 in RAW264.7 cells after incubation with various nanoparticles for 2 h. (b) The expression of TNF-α and IL-6 in RAW264.7 cells after tsMIP treatment at different concentrations, with or without chloroquine. (c) The expression of TNF-α and IL-6 in RAW264.7 cells, following tsMIP treatment, with or without chloroquine, at different time intervals. (d) Schematic representation of downstream phosphorylation inhibition. (e) Immunoblots showing phosphorylation of STAT3 (p-STAT3), phosphorylation of NF-κB p65 (p-NF-κB p65), as well as total STAT3 and NF-κB p65 expression in RAW264.7 cells treated with different NPs. GAPDH was used as a loading control.

Next, the targeted degradation of extracellular cytokines, including soluble forms of TNF-α and IL-6, was evaluated using western blotting. First, the capture and enrichment of IL-6 and TNF-α within cells were confirmed. RAW264.7 cells were stimulated with LPS for 12 h, followed by incubation with various nanoparticles for 2 h. The cells were then lysed for western blot analysis. As shown in Fig. 5a and S14, the IL-6 and TNF-α levels increased to a certain extent after LPS stimulation (Lane 1 and 6), although the enrichment is limited due to extensive extracellular secretion. Notably, the IL-6 and TNF-α levels in the tsMIP treatment group (Lane 5) were significantly higher compared to the LPS-only group (Lane 1) and other control groups (Lane 2–4), indicating successful capture and enrichment of IL-6 and TNF-α by tsMIP.

Then, the degradation of the enriched cytokines was assessed. As shown in Fig. 5b, S15 and S16, higher concentrations of tsMIP (Lane 1 and 5) induced greater degradation efficiency compared to lower concentrations (Lane 2 and 6), likely due to the increased availability of binding cavities for cytokines and IGF2R. After treatment with 100 μg mL^−1^ tsMIP for 12 h, the intracellular levels of TNF-α and IL-6 relative to those in cells where the lysosome function was inhibited by the lysosome inhibitor chloroquine were decreased by 36% and 77%, respectively. Chloroquine, a known lysosome inhibitor, partially inhibits lysosomal function,^42^ so the actual degradation efficiency might be even higher.

The degradation kinetics over time were also investigated. As shown in Fig. 5c, S17 and S18, after LPS stimulation for 12 h and incubation with tsMIP (100 μg mL^−1^) for 2 h, the intracellular IL-6 and TNF-α levels were significantly elevated (Lane 1 and 6). Following 12 h degradation, at least 26% of TNF-α and 55% of IL-6 were degraded compared to the cells treated with chloroquine. After extending the degradation period to 24 h, at least 55% of TNF-α was degraded, and IL-6 degradation reached a plateau. Since IL-6 is a secretory cytokine and TNF-α exists partially in a transmembrane form, the capture and degradation of IL-6 is more rapid than that of TNF-α. Taken together, tsMIP demonstrated high degradation efficiency towards both IL-6 and TNF-α, showing effective degradation of secretory proteins and membrane proteins simultaneously.

Upon cytokine binding to its receptor, the corresponding downstream signaling pathways are activated. Hence, we also investigated whether the reduction of cytokine levels would decrease the expression of key proteins in the associated downstream signaling pathways. To this end, we examined the IL-6–STAT3 and TNF-α–NF-κB axes. Upon cytokine–receptor interaction, the downstream proteins become phosphorylated. As shown in Fig. 5e and S19, LPS stimulation led to significant phosphorylation of STAT3 and NF-κB p65 compared to the unstimulated control (Lane 1 and 4). However, after incubation with tsMIP for 24 h, the phosphorylation of these downstream proteins was significantly decreased (Lane 5). Notably, MIP_IL-6&TNF-α_ treatment also led to some inhibition of phosphorylation (Lane 6), likely due to the steric effect. MIP_IL-6&TNF-α_ effectively binds to both cytokines, preventing their interaction with corresponding receptors. In contrast, MIP_IGF2R_ and NIP treatment did not reduce the cytokine levels nor block the downstream signaling pathways, as evidenced by the high phosphorylation level. Overall, the tsMIP not only stimulates effective cytokine degradation but also inhibits downstream signaling pathways, highlighting its potential for synergistic anti-inflammatory therapy.

### Therapeutic efficacy of tsMIP in an LPS-induced ALI mouse model

ALI is characterized by an inflammatory microenvironment, primarily driven by infiltrating immune cells and the release of pro-inflammatory mediators.^43^ Degrading the overexpressed pro-inflammatory cytokines (cytokine storm) is an effective strategy for mitigating ALI. Based on the rational design and *in vitro* results shown above, targeting key cytokines involved in cytokine storms for lysosome degradation could significantly alleviate the inflammatory state. To further validate this synergistic anti-inflammation effect *in vivo*, an LPS-induced ALI mouse model, as a typical example, was established and treated with different nanoparticle formulations. The 24-hour time point was used in this model because it is optimal for assessing LPS-induced ALI to capture the peak inflammatory response, providing a comprehensive view of the acute phase of injury. Given the typical resolution of inflammation after 24 h, this time point is often sufficient for addressing key experimental questions.^44–46^ And to maximize drug utilization, minimize dosage, reduce systemic side effects, and enable non-invasive administration,^47,48^ intranasal inhalation was chosen as the route of tsMIP delivery.

Before the verification, the *in vivo* biosafety of tsMIP was thoroughly evaluated. As shown in Fig. S20, after inhalation of a series of doses of tsMIP (0, 2, 5 and 10 mg kg^−1^), major organs, including heart, liver, spleen, lung and kidney, showed normal physiological characteristics, similar to the control group. Additionally, blood and hepatorenal function analyses of tsMIP-treated mice exhibited no abnormality and fell within the normal range, comparable to the control group (Fig. S21). Of note, the composition of other nanoparticles, including MIP_IL-6&TNF-α_, MIP_IGF2R_ and NIP, is similar to tsMIP, with the only difference being the imprinted cavities on the surface. They are all high-molecular polymers formed by hydrolysis and polymerization of silanization reagents. Hence, the biosafety of control nanoparticles was not investigated individually since similar *in vivo* biocompatibility has been widely investigated.^49–51^

After completing the biosafety assessment, the biodistribution of MIP_IGF2R_, MIP_IL-6&TNF-α_ and tsMIP was evaluated following intranasal administration in ALI mice. The ALI model was induced by intranasally administration of 10 mg kg^−1^ LPS to 6–8-week-old BALB/c mice. MIP nanoparticles were labeled with the near-infrared fluorescent dye NIR797 to track their *in vivo* distributions. As shown in Fig. S22, the MIP nanoparticles exhibited a strong near-infrared fluorescence signal after NIR797 labeling. Upon inhalation, MIP_IL-6&TNF-α_ and tsMIP accumulated more in the lungs compared to MIP_IGF2R_ and remained in the respiratory system for at least 24 h (Fig. S23 and S24), likely due to their abundant cytokine-binding cavities. At 6 h post inhalation, the nanoparticles began appearing in the liver, indicating their metabolism pathway. Taken together, both MIP_IL-6&TNF-α_ and tsMIP remained in the respiratory tract for up to 24 h, providing long-lasting synergistic anti-inflammatory effects.

**Fig. 6:**
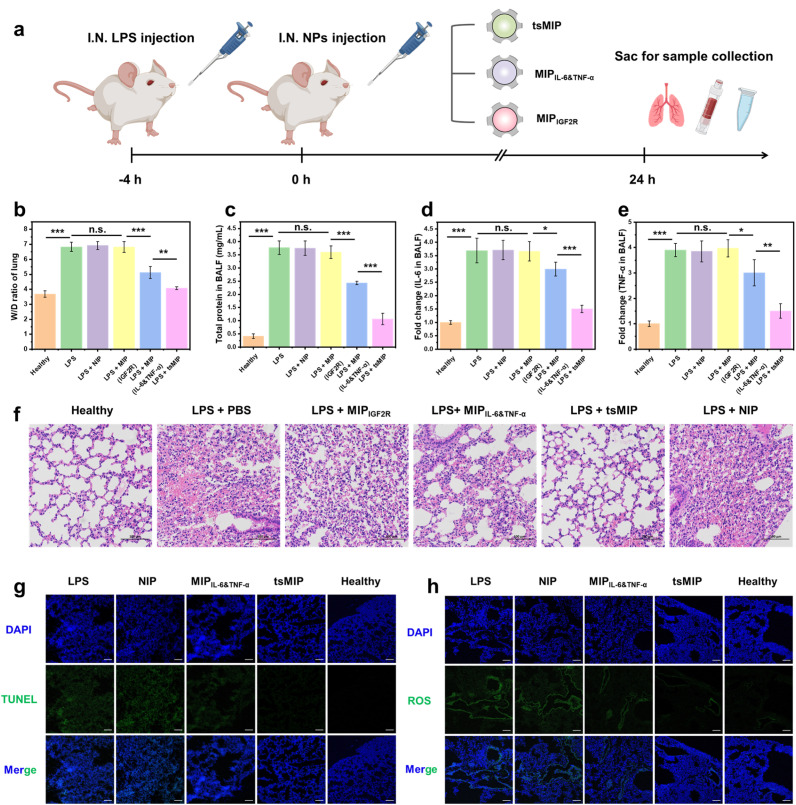
*In vivo* anti-inflammation assay using a mouse ALI model. (a) Schematic illustration of the *in vivo* anti-inflammation assay. (b) Lung wet/dry (W/D) ratios across different groups. (c) Total protein levels in mouse BALF from different groups. (d) Fold change of IL-6 secretion in BALF across different groups. (e) Fold change of TNF-α secretion in BALF across different groups (*n* = 4 for all experiments mentioned above). (f) H&E-stained lung tissue sections following the indicated treatments (scale bars, 100 μm). (g) TUNEL assay showing cell apoptosis in lung sections of all groups: TUNEL (green) and DAPI (blue). Scale bar, 100 μm. (h) Immunofluorescence staining for ROS detection in mouse lung sections: DCFH-DA (green) and DAPI (blue). Scale bar, 100 μm.

Next, the *in vivo* therapeutic effects of the nanoparticles were evaluated. As the same as above, 6–8-week-old BALB/c mice were intranasally administered 10 mg kg^−1^ LPS to induce the ALI model (Fig. 6a). After LPS stimulation for 4 h, 5 mg kg^−1^ doses of NIP, MIP_IGF2R_, MIP_IL-6&TNF-α_ and tsMIP nanoparticles dispersed in PBS solution were delivered intranasally. At 24 h post nanoparticle inhalation, the mice were sacrificed for sample collection, including bronchoalveolar lavage fluid (BALF), lung tissue, and serum. Furthermore, the wet/dry (W/D) ratios of lung tissue, total protein, cytokines including IL-6 and TNF-α, as well as inflammatory cells in BALF, were measured. As shown in Fig. 6b, mice treated with tsMIP nanoparticles exhibited significantly reduced pulmonary edema and a normal W/D ratio, comparable to healthy controls. Not only the target cytokines and total protein levels (Fig. 6c–e) but also the inflammatory cells in BALF were decreased (Fig. S25), indicating significant alleviation of the inflammatory infiltration in the lungs after tsMIP treatment. HE staining further confirmed that cytokine binding and degradation alleviated the alveolar wall thickening, alveolar cavity disappearance, and vascular congestion (Fig. 6f). Additionally, the terminal deoxynucleotidyl transferase-mediated dUTP nick-end labeling (TUNEL) staining and ROS immunofluorescence staining revealed that the targeted cytokine degradation reduced the apoptosis and ROS generation in lung tissue (Fig. 6g and h).

Notably, MIP_IL-6&TNF-α_-treated mice also showed some reduction in inflammation (Fig. 6b–f), consistent with the observed inhibition of downstream protein phosphorylation described above. This inflammation inhibition effect likely results from the interference of MIP_IL-6&TNF-α_ with cytokine–receptor interactions. Taken together, both MIP_IL-6&TNF-α_ and tsMIP demonstrated synergistic anti-inflammatory effects in the ALI mice, with tsMIP showing superior efficacy and greater potential for treating other inflammatory diseases.

## Conclusions

In this study, for the first time, we designed and developed a triple-specific molecular imprinted polymer-based LYTAC degrader to target inflammatory cytokines and lysosome receptor IGF2R simultaneously for the degradation of two key cytokines involved in CRS. The MIP-based LYTAC is particularly advantageous over antibodies for creating multivalent and multi-specific modules without the need for long-cycle biomolecular processes and complex protein expression. Owing to its rational design and engineering, the tsMIP-based LYTAC exhibits excellent cytokine capture efficiency, multi-specific targeting, robust cellular uptake, and effective lysosomal targeting for degradation. In particular, it enables the simultaneous degradation of secretory proteins and membrane proteins, which is difficult for the traditional LYTAC. After the degradation of target cytokines, the corresponding downstream signal pathways, including IL-6–STAT3 and TNF-α–NF-κB axes, are effectively inhibited, verifying its comprehensive synergistic anti-inflammatory effect, which was further confirmed by *in vivo* experiments in the ALI Balb/c mouse model as well. Overall, the tsMIP-based LYTAC we developed provides a promising new strategy for treating CRS and has the potential to be expanded to other inflammatory diseases. In future work, this approach could be adapted to target other difficult-to-drug biomolecules for multi-specific degradation relying on the designable multi-module of MIP nanoparticles, providing a versatile platform with great potential for clinical translation.

## Ethical statement

All the animal experiments were performed in accordance with the ethical guidelines approved by the Animal Management and Ethics Committee of Nanjing University (220200451).

## Author contributions

J. Chen, W. Lu and Z. Liu designed the project. J. Chen performed the bioinformatics analysis, *in vivo* experiments and part of the *in vitro* experiments. W. Lu synthesized the nanomaterials and performed the *in vitro* experiments. J. Chen and W. Lu contributed equally to this work. Y. Li and Z. Guo performed TEM characterization and partial nanomaterials synthesis. Q. Liu and W. Liu performed the circular dichroism analysis. J. Chen drafted the manuscript. L. Wang commented and revised the manuscript. Z. Liu finalized the manuscript and supervised the whole project. All authors approved the final version of the manuscript.

## Conflicts of interest

There are no conflicts to declare.

## Supplementary Material

SC-OLF-D5SC04757A-s001

## Data Availability

Original data are available upon request from the corresponding authors with an appropriate reason. All data supporting the findings of the work are provided in the manuscript and the supplementary information (SI). Supplementary information is available: includes additional descriptions of experimental procedures and instruments, as well as supporting figures. See DOI: https://doi.org/10.1039/d5sc04757a.
