# Supplementary material for: Tri-specific molecularly imprinted lysosomal nanodegrader enables synergistic therapy of cytokine storm
Source: Chem Sci. 2025 Sep 23;16(42):20002–11. doi: 10.1039/d5sc04757a (PMC12481441; doi:10.1039/d5sc04757a)
Supplement: SC-016-D5SC04757A-s001 [file SC-016-D5SC04757A-s001.pdf]

Supplementary Information (SI) for Chemical Science.
This journal is © The Royal Society of Chemistry 2025

Supplementary Information

Tri-specific molecularly imprinted lysosomal nanodegrader enables synergistic therapy of cytokine storm

Jingran Chen,[a, †] *Weihua Lu,*[a, †] *Ying Li,*[a] *Zhanchen Guo,*[a] *Qian Liu,*[a] *Weiwei Liu,*[a] *Lisheng Wang,*[b] *and Zhen Liu*[a,*]

[a] State Key Laboratory of Analytical Chemistry for Life Science, School of Chemistry and Chemical Engineering, Nanjing University, Nanjing 210023, China

[b] Department of Biochemistry, Microbiology and Immunology, Faculty of Medicine, University of Ottawa, Ottawa, Ontario K1H 8M5, Canada

† J. Chen and W. Lu contributed equally to this work.

* Corresponding author: zhenliu@nju.edu.cn

Experimental Section

1. Reagents and materials. Benzyltriethoxysilane (BnTES) and ammonia (28%) were purchased from Sinopharm Chemical Reagents (Shanghai, China). 3-Aminopropyltriethoxysilane (APTES, 98%), isobutyltriethoxysilane (IBTES, 98%), 3-ureidopropyl-triethoxysilane (UPTES, 50%) and tetraethyl orthosilicate (TEOS, 99%) were purchased from J&K Scientific (Shanghai, China). C13-modified VTLRSTRQT, QVYFGVIAL, and QAVDLDALCK (HPLC verified, purity above 98%) were synthesized by Top-Peptide Biotechnology (Shanghai, China). Acetic acid (HAc) and absolute ethanol were purchased from Nanjing Chemical Reagent Co. (Nanjing, China). N-hydroxylsuccinimide-polyethylene glycol (mPEG-NHS) (MW: 1000 Da) was purchased from Aladdin Reagents (Shanghai, China). 3-(4,5-Dimethylthiazole-2)-2,5-diphenyltetrazolium bromide (MTT), Lyso-Tracker Red, CellTiter Lumi™ Stable Luminescent Cell Viability Assay Kit, RIPA (Radio Immunoprecipitation Assay) Lysis Buffer, Phenylmethylsulfonyl fluoride (PMSF), BeyoGel™ Plus precast gel (4-20%), 5×SDS protein loading buffer, BeyoECL Star and QuickBlock™ immunoblotting solution set were obtained from Beyotime (Shanghai, China). Lipopolysaccharide (LPS) was obtained from Sangon biotech (Shanghai, China). Tricolor prestained protein molecular weight standards (8–180 kDa) and goat anti-rabbit IgG H&L (Alexa Fluor® 488) were purchased from Yeasen Biotechnology (Shanghai, China). DMEM high-glucose medium, phosphate buffered saline solution (1×PBS) for cell culture, and BCA protein detection kit were purchased from Keygen Biotech (Nanjing, China). Fetal bovine serum (FBS) was purchased from Gibco (Life Technologies, Australia). Anti-GAPDH rabbit monoclonal antibody (ab181602), anti-IGF2R rabbit monoclonal antibody (ab150079), anti-IL-6 rabbit monoclonal antibody (ab290735), anti-TNF-α rabbit monoclonal antibody (ab183218), goat anti-rabbit IgG (H L) HRP (ab205718), and goat anti-rabbit IgG H&L (Alexa Fluor® 647) (ab150079) were purchased from Abcam Biosciences (Shanghai, China). Deionized water used in all experiments was purified by a Milli-Q Advantage A10 ultrapure water purification system (Millipore, Milford, MA, USA).

2. Instruments. Transmission electron microscopy (TEM) was performed on ahigh-resolution transmission electron microscope (JEOL, Tokyo, Japan), operating at a voltage of 200 kV. Ultraviolet-visible (UV) spectral analysis was performed using a NanoDrop 2000/2000C spectrophotometer (Thermo Fisher, MA, USA). Fluorescence intensity was measured using a BioTek Synergy Mx microplate reader (Winooski, VT, USA). Particle size distribution and zeta potential measurements were performed on a BI-200SM instrument (Brookhaven, Holtsville, NY, USA). Confocal fluorescence imaging was performed on a LSM 710 confocal laser scanning microscope (Zeiss, Oberkochen, Germany). Gel electrophoresis was performed on a Mini-PROTEAN® Tetra Cell system (Bio-Rad, California, USA), and immunoblot imaging was performed on a Tanon-4600SF gel imager (Tanon, Shanghai, China). Flow cytometry was conducted on a Beckman Coulter CytoFlex S system (California, USA).

3. Cell culture. RAW264.7 was cultured in DMEM medium with 10% fetal bovine serum (37 °C, 5% CO2).

4. Molecular Docking. Molecular docking was performed by autodock vina [1, 2]. Three epitopes were added to the system and the optimal structure is selected based on the docking score and the conformation rationality. The docking results were visualized using PyMOL software.

5. Circular dichroism spectroscopy. First, individual peptide solutions of IL-6, TNF-α, and IGF2R were prepared at a concentration of 100 μM. Subsequently, a mixed solution containing all three peptides was prepared, with each peptide maintained at a final concentration of 100 μM, followed by incubation under gentle agitation on a thermomixer at room temperature for 1 h. Circular dichroism (CD) spectra were then recorded over the wavelength range of 180–260 nm with a step size of 1 nm. Each experiment was conducted three times, and the background signal from the buffer was subtracted. The theoretical additive spectrum was obtained by weighted summation of the individual CD spectra of the three peptide solutions.

5. Bioinformatics analysis. RNA-Seq datasets (GSE216645, GSE216943 and GSE263867) were downloaded from the Gene Expression Omnibus (GEO), and then differentially expressed genes (DEGs) were identified using the R programming language. Among the 443 overlapping genes, 378 genes were found significantly upregulated, while 64 downregulated (Figure S1a-c, Figure S2). These DEGs were further analyzed using the Gene Ontology (GO) and Kyoto Encyclopedia of Genes and Genomes (KEGG) databases, which revealed the gene interaction and corresponding signal pathways. A comprehensive protein-protein interaction (PPI) network was then constructed, with blue notes representing downregulated genes and red notes representing upregulated genes (Figure S3a). Hub genes within the PPI network were identified using different algorithms (Figure S1f), including closeness, degree, stress, edge percolated component (EPC), maximum neighborhood component (MNC), and maximal clique centrality (MCC), with their upregulated intersections displayed in a venn diagram. As shown in Figure S3b, cytokines including IL-6 and TNF family, as well as chemokines including CCL2 and CXCL10 are upregulated.

6. The preparation of tsMIP. TsMIP was synthesized according to the reverse microemulsion imprinting approach previously reported by our group [3] with appropriate modifications, with its surface functionalized with PEG after imprinting.[4] In order to be compatible with the reversed-phase microemulsion system, the selected peptides need to be grafted with a hydrophobic fatty acid chain to obtain an amphiphilic template. For C-terminal nonapeptide, the tridecanoic acid was directly grafted on the N-terminal. For N-terminal nonapeptide, a lysine (K) was first introduced to the C-terminal of the epitopes and then grafted with tridecanoic acid. In detail, the ε-amino group of lysine was protected with Dde, while the α-amino group of the backbone was protected with Fmoc. The Dde group was selectively removed using hydrazine, enabling the conjugation of C13 to the lysine side chain. Finally, the Fmoc group was removed with piperidine, ensuring that C13 was exclusively attached to the lysine side chain rather than the backbone amino group. The four silanization reagents interact with amino acids of different properties, with their specific structures shown in Figure S2. APTES with amino groups can interact with acidic amino acids (group I) through electrostatic attraction or with other amino acids in group V through hydrogen bonds; UPTES with carbamide groups can interact with basic amino acids (group II) and

other amino acids in group V through hydrogen bonds; IBTES with hydrophobic groups can interact with hydrophobic amino acids (group IV) through hydrophobic interactions; BnTES with phenyl groups can interact with aromatic amino acids (group III) through π-π stacking. TEOS was used as a cross-linker to form a silica skeleton during the imprinting process. The ratio of TEOS: functional monomer was determined to be 7:3 in this work.

6.1 Microemulsion formation along with template anchoring. 1.77 g of Triton X-100, 6.5 mL of cyclohexane, 1.6 mL of n-hexanol, 100 μL of ammonium hydroxide, and 480 μL of water were added to a 25-mL eggplant flask with a 1.5-cm magnetic stir bar. The mixture was stirred at 700 rpm for 30 min at room temperature to form a clear and transparent solution. Then 1 mg of each amphiphilic template C13-VTLRSTRQT, C13-QVYFGVIAL and QAVDLDALCK-C13 was added into the evenly mixed system and continue stirring for 20 min.

6.2 Reverse microemulsion-confined interface imprinting. First, solution A and solution B were prepared for subsequent dripping into the reverse microemulsion system. 100 μL of silanization reagent mixed solution containing a specific APTES/UPTES/IBTES/BnTES ratio was added into 1 mL of cyclohexane, followed by evenly mixing to obtain solution A. Then, 100 μL of TEOS was added to 1 mL of cyclohexane and mixed evenly to obtain solution B. 300 μL of solution A and 700 μL of solution B were mixed evenly to obtain solution X. Subsequently, 1 mL of solution X was slowly added dropwise to the stable microemulsion system, and stirred at 700 rpm for 24 h at room temperature. After that, 20 μL TEOS/APTES (V:V = 5:3) solution was added and stirred at room temperature for 12 h.

6.3 Microemulsion disruption and surface PEGylation After imprinting, the prepared tsMIP nanoparticles were released from the emulsion by adding 6 mL of acetone, then the mixture was centrifuged for 20 min at 8,000 rpm in order to separate the tsMIP from the suspension. The obtained nanoparticles were washed by ethanol and deionized H_2O successively for three times, and lyophilized overnight. After that, 10 mg of tsMIP nanoparticles were dispersed in 1 mL H_2O, followed by addition of 3 mg mPEG-NHS and 170 μL PBS (1×). The mixture was vigorous shaken at room temperature for 24 h, then the mixture was centrifuged at 8,000 rpm for 20 min to collect the prepared tsMIP-PEG

nanoparticles. In the end, the nanoparticles were washed by ethanol and deionized H_2O successively for three times.

6.4 Template Removal. The obtained nanoparticles were dispersed into 20 mL of mixed solution of acetonitrile, H_2O, and acetic acid (v: v: v = 50: 49: 1) and shaken at room temperature for 2 h. Then the solution was centrifuged at 8,000 rpm for 20 min to collect the prepared tsMIP-PEG. The obtained nanoparticles were washed by ethanol and deionized H_2O successively for three times and then stored at 4 °C for later use.

For the preparation of FITC-dopped tsMIP, the procedure was the same as above except that 2 mg of FITC and 20 μL of APTES were introduced after the template addition.

For the preparation of non-imprinted polymer (NIP), the procedure was the same as above except that the template addition was omitted.

For the preparation of single template (IGF2R)-imprinted MIP and dual template (IL-6 and TNF-α)-imprinted MIP, the procedure was the same as above except that the corresponding templates were added.

7. Optimization of monomer composition. First, 0.1 mg/mL solutions of C-terminal nonapeptide epitope of IL-6 (VTLRSTRQT), C-terminal nonapeptide epitope of TNF-α (QVYFGVIAL) and N-terminal nonapeptide epitope of IGF2R (QAVDLDALC) were prepared separately using PBS (1×). Then 2 mg of tsMIP and NIP, which were prepared at different monomer ratios (APTES, UPTES, IBTES, BnTES, and TEOS), were dispersed into 200 μL of peptide solution. After incubation at room temperature for 1 h, nanoparticles were collected by centrifugation and washed three times with the PBS buffer. Subsequently, the nanoparticles were redispersed in 20 μL of mixed solution of acetonitrile, H_2O, and acetic acid (v: v: v = 50: 49: 1) and shaken at room temperature for 10 min. Finally, the eluate was collected by centrifugation. The amount of C-terminal epitope of IL-6, C-terminal epitope of TNF-α, and N-terminal epitope of IGF2R captured by tsMIP or NIP were determined by measuring the absorbance of elutes at 214 nm. Three parallel samples were set for each group. The imprinting factor (IF) was calculated based on the ratio of the absorbance of the eluate in the tsMIP group to the absorbance of the eluate in the NIP group. In this way, the imprinting effect was evaluated and the monomer ratio was optimized.

8. Selectivity test of tsMIP. APMAEGGGQ, EDPQGDAAQ, GRVVNPTQK, and KSLLSPGK were used as test peptides to evaluate the selectivity of tsMIP on the C-terminal nonapeptide epitope of IL-6 (VTLRSTRQT), the C-terminal nonapeptide epitope of TNF-α (QVYFGVIAL), and N-terminal nonapeptide epitope (QAVDLDALC) of IGF2R at the peptide level. Various peptide solutions (0.1 mg/mL) were prepared by PBS buffer (1×). 2 mg of tsMIP and NIP were dispersed in the peptide solution and incubated at room temperature for 1 h. Then the solution was centrifuged and the nanoparticles were washed three times with PBS buffer (1×). Finaly, tsMIP and NIP were redispersed in 20 μL of mixed solution of acetonitrile, H_2O, and acetic acid (v: v: v = 50: 49: 1) and shaken at room temperature for 10 min. The eluates were collected by centrifugation and the absorbance at 214 nm was measured. The analysis was conducted for 3 independent times.

9. Determination of adsorption isotherm. A series concentration of epitope solutions including C-terminal nonapeptide epitope of IL-6 (VTLRSTRQT), C-terminal nonapeptide epitope of TNF-α (QVYFGVIAL) and N-terminal nonapeptide epitope of IGF2R (QAVDLDALC) were prepared by PBS buffer (1×). 2 mg of tsMIP nanoparticles were dispersed into 200 μL of peptide solution and incubated at room temperature for 1 h. After centrifuging, the tsMIP was collected. Then the nanoparticles were washed by PBS buffer for three times. Subsequently, tsMIP was redispersed in 20 μL of mixed solution of acetonitrile, H_2O, and acetic acid (v: v: v = 50: 49: 1) and shaken at room temperature for 10 min. The solution was centrifuged and the absorbance of the eluates were measured. Then an adsorption isotherm was established by plotting the relationship between the absorbance of the eluates and the concentration of the peptide. Hill fitting (n=1) was performed on the obtained data to calculate the affinity of tsMIP to the C-terminal epitope of IL-6, the C-terminal epitope of TNF-α, and the N-terminal epitope of IGF2R. And the saturate adsorption towards three epitopes were calculated as well, in virtue of the adsorption isotherm and the UV absorbance standard curves of three epitopes.

Hill equation:

$$Y = B_{\mathrm{max}}X^{\mathrm{n}}/(K_{\mathrm{d}}^{\mathrm{n}}+X^{\mathrm{n}})\ (\mathrm{n} = 1) \qquad (1)$$

10. Estimation of binding valency and apparent molecular mass of tsMIP. To evaluate the binding capacity of tsMIP towards three targeted epitopes, the number (N) of binding sites on each nanoparticle were calculated as the equation given below:

$$N = \frac{\frac{4}{3}\pi R^3 \rho Q_{max} N_A}{M} \qquad (2)$$

Where R is the radius of a single nanoparticle (R ≈ 15 nm), Q_{max} is the saturated adsorption amount of TNF-α, IL-6 and IGF2R epitopes bound by tsMIP particles, estimated by corresponding UV absorbance value (standard curves and binding isotherm of three epitopes), ρ is the density of silica (ρ = 2.2 g cm^{-3}), N_A = 6.02 × 10^{23} mol^{-1}, $M_{TNF\text{-}\alpha}$ = 1009.19 Da, $M_{IL\text{-}6}$ = 1061.19 Da, M_{IGF2R} = 947.06 Da.

The apparent molar mass of tsMIP was estimated according to the equation below:

$$M_{tsMIP} = \frac{4}{3}\pi R^3 \rho N_A \qquad (3)$$

Considering the mean diameter of tsMIP was 30 ± 5.4 nm (Figure S6), the apparent molar mass of tsMIP was estimated to be around 18,700 kDa.

11. Stability of tsMIP stored in PBS. In order to evaluate the storage stability of tsMIP in PBS solution, the particle size of which was monitored within 7 days. The 10 mg/mL tsMIP stock solution was diluted to 0.5 mg/mL with PBS buffer, and then the particle size distribution was measured using DLS particle size analyzer on days 0, 3, and 7 respectively.

12. Cytotoxicity of tsMIP. RAW264.7 cells were first seeded on a 96-well microplate at a density of 10^4 per well. After seeding, the cells were cultured for 12 h for cells adhering. For cytotoxicity analysis of tsMIP and NIP, the pre-seeded RAW264.7 cells were incubated with different concentrations of tsMIP or NIP for 24 h. The wells without nanoparticles were used as the control group. The wells without cells were used as the background group. All other wells were set as test groups. Then the cell viability was evaluated by MTT assay. In detail, cell viability is expressed as the percentage of the absorbance of the test cells to the control cells (both were subtracted from the background absorbance), and the calculation formula is as follows:

$$\text{Cell viability (\%)} = \frac{\text{Abs (test)-Abs (background)}}{\text{Abs (control)-Abs(background)}} \times 100\% \quad (4)$$

The analysis was conducted for 3 independent times.

13. Cell uptake experiment. First, RAW264.7 cells were incubated with 1 μg/mL LPS for 12 h to stimulate the secretion of pro-inflammatory cytokines as an in vitro inflammation model. Then, 100 μg/mL of FITC-doped NIP, tsMIP, MIP_{IGF2R} and $MIP_{IL-6\&TNF-\alpha}$ were incubated with cells separately at 37 °C for 2 h. Subsequently, the cells were washed by PBS (1×) for two times and redispersed in PBS (1×) at the density of 5×10^6 cells/mL. After being filtered by 200-mesh cell sieve, the cells were analyzed by flow cytometry.

14. Degradation degree analysis. In the analysis of the degradation degree of transmembrane TNF-α (tmTNF-α), RAW264.7 cells were first stimulated by 1 μg/mL LPS and incubated with a certain concentration of nanoparticles for a certain period of time. Then the cells were washed twice with PBS (1×), and collected by centrifugation at 1,000 rpm for 4 min. After that, the cells were fixed with 200 μL 4% paraformaldehyde for 15 min at room temperature and blocked with 5% BSA for 1 h. Subsequently, the cells were incubated with 200 μL of 1:5,000 diluted anti-TNF-α antibody for 1 h and rinsed with 200 μL PBS. Finally, the cells were incubated with 200 μL of 1:200 diluted Alexa Fluor® 488-labeled goat anti-rabbit IgG (H+L) antibody for 30 min, washed with PBS and then redispersed in 200 μL PBS. Cells only stimulated by 1 μg/mL LPS without nanoparticles incubation were set as blank group. All the measurements were performed by flow cytometry within 1 h and the quantitative analysis were conducted on FlowJo software.

15. Lysosomal colocalization assay. RAW264.7 cells were first seeded onto confocal culture dish for adhesion and then stimulated with 1 μg/mL LPS for 12 h. After that, the pretreated cells were incubated with 100 μg/mL FITC-doped nanoparticles for 2 h. Subsequently, cells were washed twice and incubated with Lyso-Tracker Red for 10 min at 37 °C. Finally, the cells were washed with PBS solution (1×) and imaged on LSM 710 confocal laser scanning microscope.

16. Cytokine capture assay. Firstly, RAW 264.7 cells were stimulated by 1 μg/mL LPS. Then the cell culture medium was collected to incubate different nanoparticles. 100 μg/mL of tsMIP and NIP nanoparticles for different incubation time or various concentration of tsMIP and NIP for 2 h incubation were labeled by primary antibody. Then the complex was incubated with Goat anti-rabbit IgG H&L (Alexa Fluor® 647) secondary antibody for quantitative analysis. After centrifugation and being redispersed in 100 μL of PBS solution (1×), the fluorescence intensity was measured in 96-well plate to evaluate the level of cytokines captured by the particles.

17. Immunofluorescence staining. RAW264.7 cells were first stimulated by 1 μg/mL LPS for 12 h, then treated with 100 μg/mL tsMIP, NIP, MIP_{IGF2R} or $MIP_{IL\text{-}6\&TNF\text{-}\alpha}$ for 12 h. After that, the cells were fixed with 4% paraformaldehyde for 10 min and blocked for 1 h in PBS containing 10% FBS (v/v) and 5% BSA (w/v). Subsequently, the cells were rinsed by PBS solution and incubated with anti-TNF-α antibody for 2 h at room temperature, and then labeled by AlexaFluor® 488 goat anti-Rabbit IgG for 1 h. Finally, the cells were incubated with DAPI for 5 min and sent to CLSM imaging.

18. Western blot assay. As for the capture and enrichment of IL-6 and TNF-α within cells, RAW264.7 cells were first stimulated by LPS for 12 h, followed by incubation with various nanoparticles for 2 h. As for degradation efficiency assessment, cells were treated with 100 μg/mL tsMIP for 12 h. As for degradation kinetics over time, cells were stimulated by LPS for 12 h and incubated with tsMIP (100 μg/mL) for 2 h. After the pretreatments, the cells were then lysed for western blot analysis. Firstly, the cells were lysed with RIPA lysis buffer which containing protease inhibitors, phosphatase inhibitors and EDTA for 8 min on ice. Then the lysate was centrifuged at 10,000 g for 10 min at 4 °C, and the protein concentration was determined by BCA assay. Subsequently, 5×SDS PAGE protein loading buffer were added to the lysate and denatured at 95 °C for 10 minutes. Lysates containing the same amount of total protein (20-50 μg protein) were loaded onto a 4-20% Tris-Gly SDS PAGE gel and run at 80 V for 30 min, followed by 120 V for 1 h. Next, the gel was transferred to a hydrophilic polyvinylidene fluoride (PVDF) membrane (0.45 μm) at a constant flow of 350 mA for 1 h. The membrane was then blocked with QuickBlock™ blocking buffer for 30 min at room

temperature, and incubated with 1:2,000 diluted anti-TNF-α antibody and 1:10000 diluted anti-GAPDH antibody overnight at 4 °C. After washing, the membrane was incubated with HRP-conjugated secondary antibodies for 1 h at room temperature, and then washed three times for chemiluminescence imaging. Finally, the protein levels were quantified by analyzing the gray value of each band using ImageJ software.

19. Animal experiments

19.1 *In vivo* biosafety experiment assay. 6-8-week-old male BALB/c mice were purchased from Vital River (Beijing, China) and maintained under controlled conditions of temperature (23 ± 2 °C), humidity (45 ± 10%) and lighting (12-h artificial light and dark cycles) with air exchange. Sterilized water and mouse food are changed regularly to ensure the health of the mice. 16 mice were randomly divided into four groups and fed in four different methyl pentene copolymer (TPX) cages for a week before biosafety evaluation. Before the nasal administration, mice were lightly anesthetized by inhaled isoflurane and placed in a supine posture. Then, tsMIP of different dosages (0, 2, 5, 10 mg/kg) were inhaled through nasal of BALB/c mice. After inhalation for 48 h, the mice were sacrificed for sample collection. The organs (heart, liver, lung, spleen and kidney) and blood samples (whole blood and serum) were harvested for analysis, including organ H&E staining, whole blood analysis and serum biochemical tests. For whole blood analysis, white blood cells (WBC), red blood cells (RBC), platelets (PLT), hemoglobin (HGB), mean corpuscular volume (MCV), mean corpuscular hemoglobin (MCH), mean corpuscular hemoglobin concentration (MCHC) and hematocrit (HCT) were investigated by automatic blood cell analyzer BC-5000vet (Mindray Animal, China). For serum biochemical analysis, the whole blood was centrifuged at 3,000 rpm for 15 min to obtain the supernatant serum samples. Then the samples were analyzed for biochemical parameters of kidney function, including blood urea (UREA), creatinine (CREA) and uric acid (UA), and liver function, including alanine aminotransferase (ALT), aspartate aminotransferase (AST) and total bilirubin (TBIL), by standard techniques using a Chemray 800 automatic biochemical analyzer (Rayto, China).

19.2 *In vivo* Biodistribution. Firstly, NIR797 was dopped into tsMIP, $MIP_{IL\text{-}6\&TNF\text{-}\alpha}$ and MIP_{IGF2R} for *in vivo* imaging. The fabrication procedure was the same as the FITC dopped MIP which was described before, except that the dye was replaced as NIR797. Before the nasal administration, mice were lightly anesthetized by inhaled isoflurane. Then the healthy 6-8 week-old female BALB/c mice were placed in supine position, and 10 mg/kg LPS was intranasally added to induce ALI model. 4 h later, the mice were anesthetized again and then different NIR797 dopped nanoparticles dispersed in 1 × PBS solution were intranasally added to mice. Subsequently, at different time intervals (0, 1, 6, 12, 24 h), the distribution of nanoparticles in mice was imaged using the PerkinElmer *in vivo* imaging system. At every time interval, one of the mice were sacrificed, and the major organs (heart, liver, lung, spleen and kidney) were harvested for *ex vivo* imaging (Figure S16).

19.3 *In vivo* Inflammation inhibition. The healthy 6-8-week-old female BALB/c mice were randomly divided into 6 groups, 4 mice in each group: Healthy, LPS, LPS+NIP, LPS+MIP_{IGF2R}, LPS+$MIP_{IL\text{-}6\&TNF\text{-}\alpha}$, LPS+tsMIP. 10 mg/kg LPS was first intranasally administrated for ALI model induction. At 4 h post the challenge, the mice in different groups received inhalation of PBS solution (1×) (Healthy), 5 mg/kg NIP nanoparticles dispersed in PBS solution (LPS+NIP), 5 mg/kg MIP_{IGF2R} nanoparticles dispersed in PBS solution (LPS+MIP_{IGF2R}), 5 mg/kg $MIP_{IL\text{-}6\&TNF\text{-}\alpha}$ nanoparticles dispersed in PBS solution (LPS+$MIP_{IL\text{-}6\&TNF\text{-}\alpha}$) and 5 mg/kg tsMIP nanoparticles dispersed in PBS solution (LPS+tsMIP). After LPS stimulation and different nanoparticles administration, the mice were sacrificed at 24 h time point for sample collection, including bronchoalveolar lavage fluid (BALF), lung tissue, and serum. Further, the wet/dry (W/D) ratio of lung tissue, total protein, cytokines including IL-6 and TNF-α, as well as inflammatory cells in BALF were measured.

For BALF collection, lung lavage was carried out by introducing 300 μL cold PBS into lungs then carefully withdrawing. The BALF was then centrifuged at 1,000 rpm for 10 min at 4 °C for cells and protein collection: lung cells in the precipitate and proteins in the supernatant. The supernatants were stored at -80 °C for cytokine analysis, and the cell pellets were first processed with red blood cell lysis buffer and then redispersed in PBS solution for cell

staining. The cell suspensions were dropped on the surface of glass slide and sit for 10 min until the cells settled on the surface due to the gravity. Then the cells were stained with Giemsa regent for cell differentiation: basophils are stained blue-purple, neutrophils are stained lavender and mononuclear cells are stained blue-purple or purple-red. Thereafter, the slides were imaged on a microscope for cell counting. The total protein and specific cytokine were analysed by BCA assay and corresponding cytokine ELISA kit according to the manufacturer's instructions.

The left larger lobe of lung tissue was conducted hematoxylin and eosin (H&E) staining for histological injures analysis, immunofluorescence staining for ROS detection and Tunel staining for apoptosis analysis. The remaining lung tissues were weighed before and after the drying process (60 °C, 48 h) for W/D ratio calculation. All the animal experiments were performed in accordance with the ethical guidelines approved by the Animal Management and Ethics Committee of Nanjing University (220200451).

20. Statistical Analysis. All data were obtained from at least three parallel experiments and expressed as mean value ± standard deviation. Two sample t-test was applied to test the significance of the difference between different groups, which was regarded to be significant when P was < 0.05 (*) and very significant when P was < 0.01 (**) or < 0.001 (***). Data are available on request from the authors.

Supplementary Figures

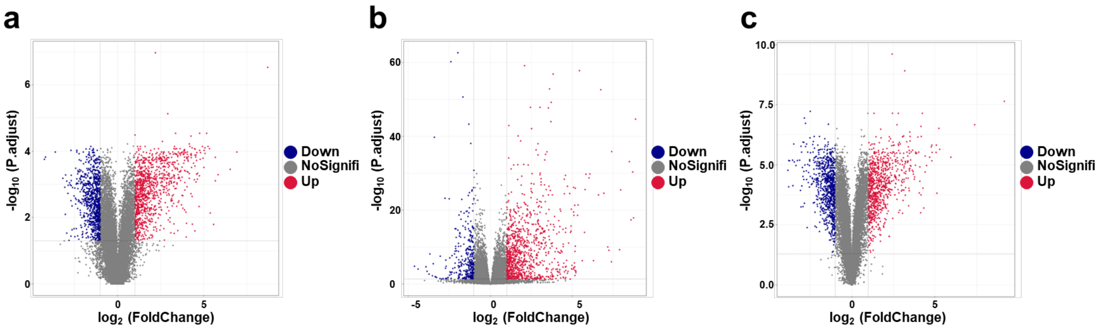

Figure S1. The differentially expressed genes in 3 RNA SEQ datasets. (a) GSE216645. (b) GSE216943. (c) GSE263867.

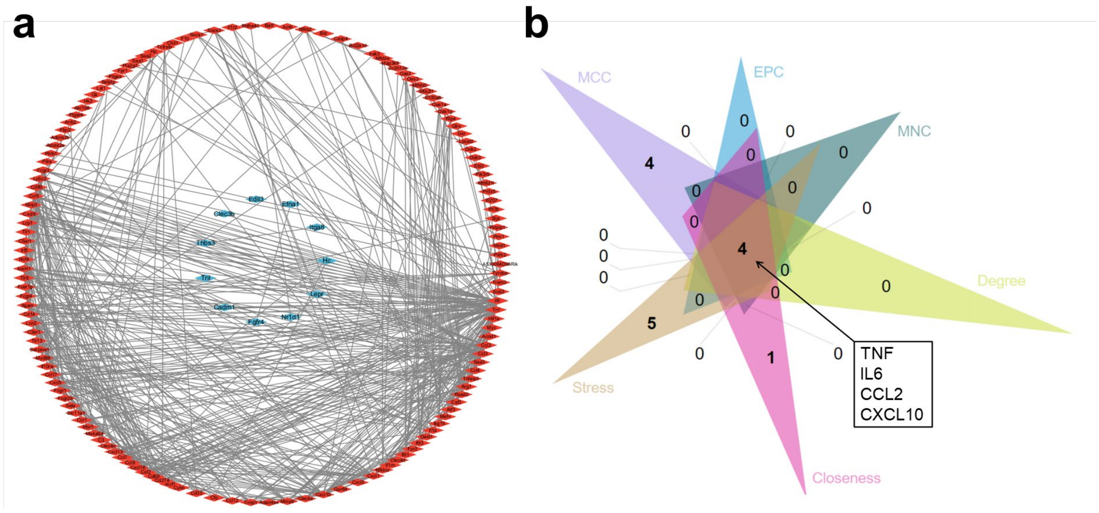

Figure S2. The PPI network (a) and up-regulated genes interactions of different algorithms (b).

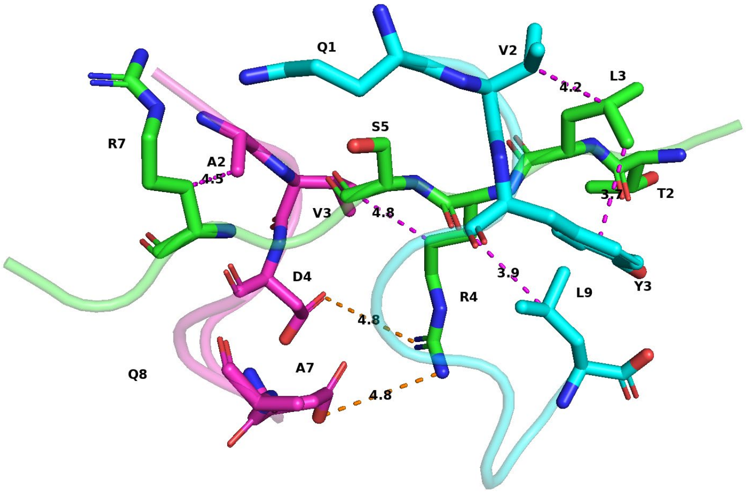

Figure S3. Interaction analysis among peptides QVYFGVIAL, VTLRSTRQT and QAVDLDALC via molecular docking. The orange dotted line is the electrostatic interaction, the magenta dotted line is the π-alkyl hydrophobic interaction.

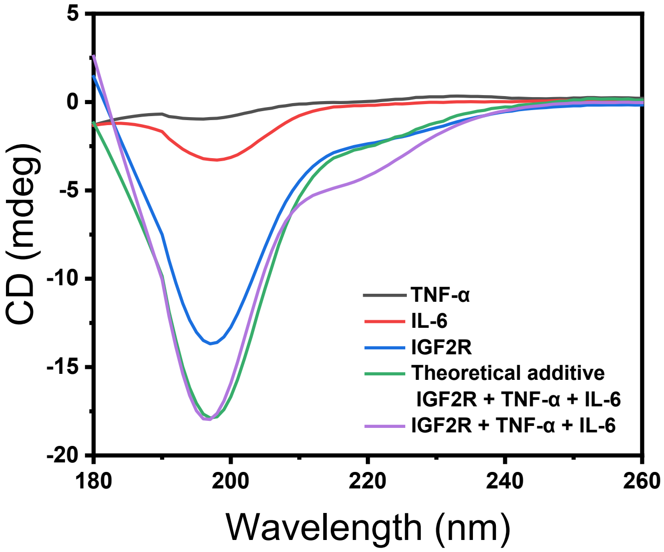

Figure S4. CD spectrum of three epitopes and their theoretical additive spectrum.

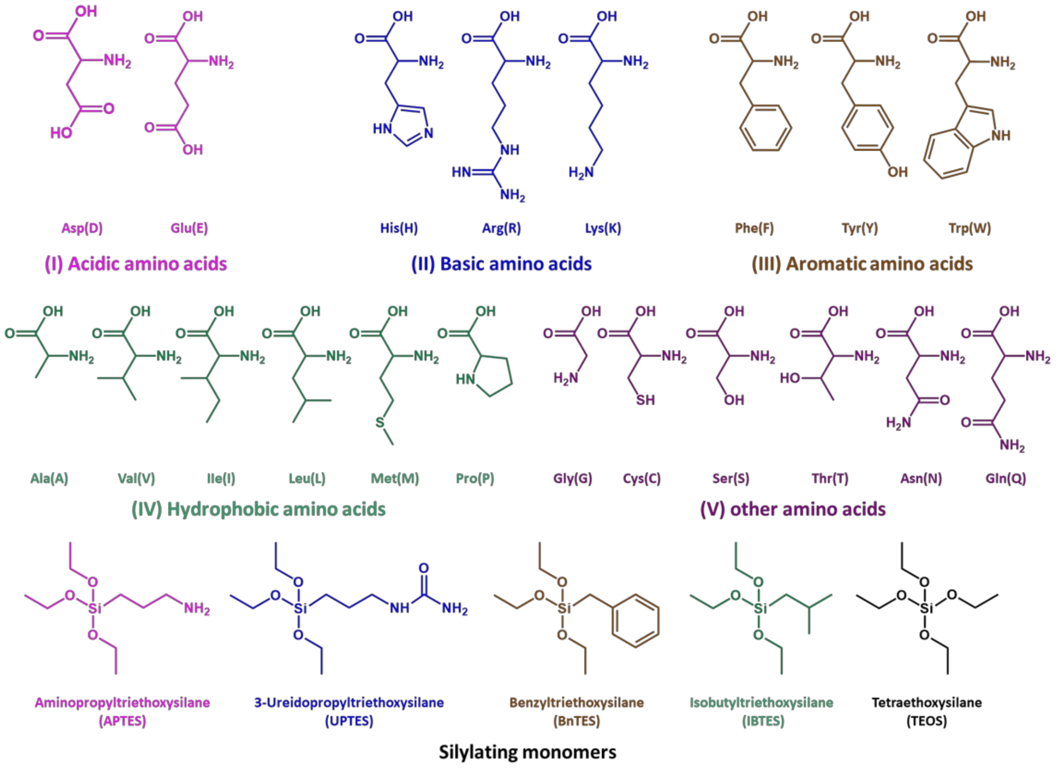

Figure S5. The classification of amino acids and structures of silylating monomers used in this study.

a VTLRSTRQT

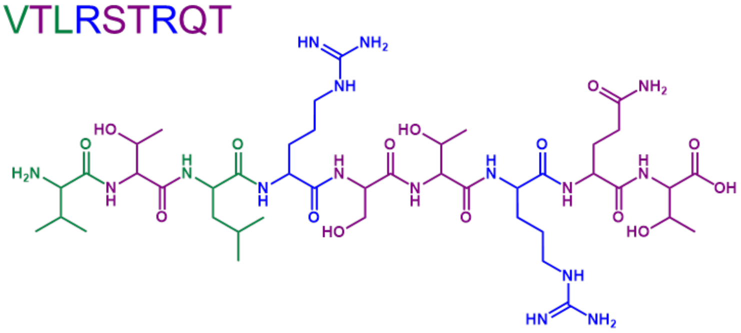

b QVYFGVIAL

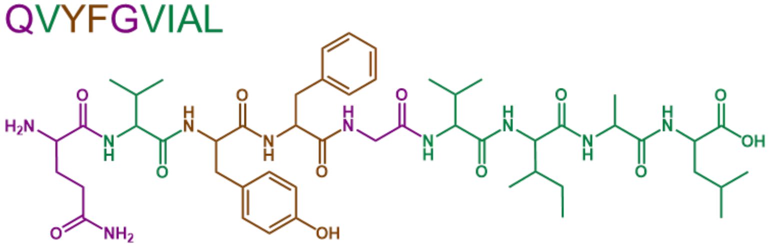

c QAVDLDALC

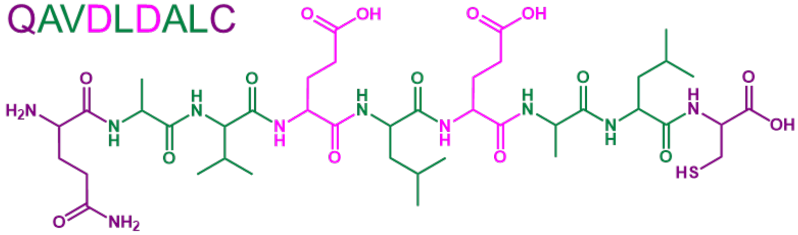

Figure S6. Structures of the epitopes used in this study. a) C-terminal epitope of IL-6; b) C-terminal epitope of TNF-α; c) N-terminal epitope of IGF2R.

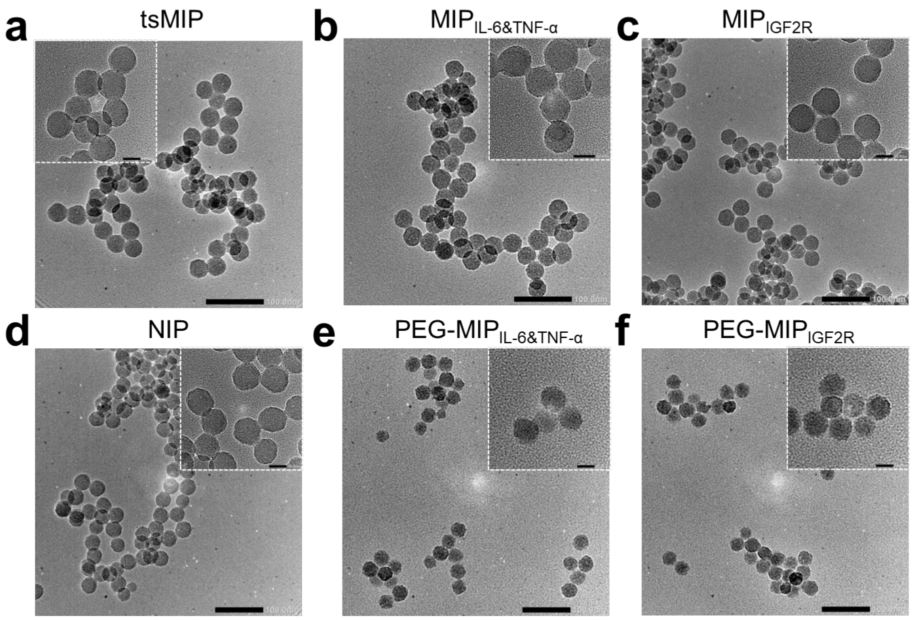

Figure S7. The TEM images of tsMIP, $MIP_{IL\text{-}6\&TNF\text{-}\alpha}$, MIP_{IGF2R} and NIP nanoparticles, as well as the PEG modified $MIP_{IL\text{-}6\&TNF\text{-}\alpha}$ and MIP_{IGF2R}. a) tsMIP; b) $MIP_{IL\text{-}6\&TNF\text{-}\alpha}$; c) MIP_{IGF2R}. d) NIP; e) PEG modified $MIP_{IL\text{-}6\&TNF\text{-}\alpha}$; f) PEG modified MIP_{IGF2R}. The enlarged images of the nanoparticles are shown within the dotted box.

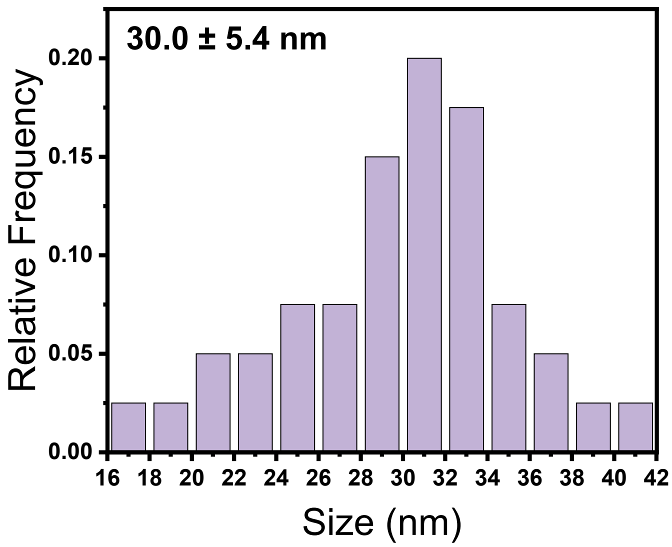

Figure S8. The diameter distribution of synthesized tsMIP analyzed by Image J.

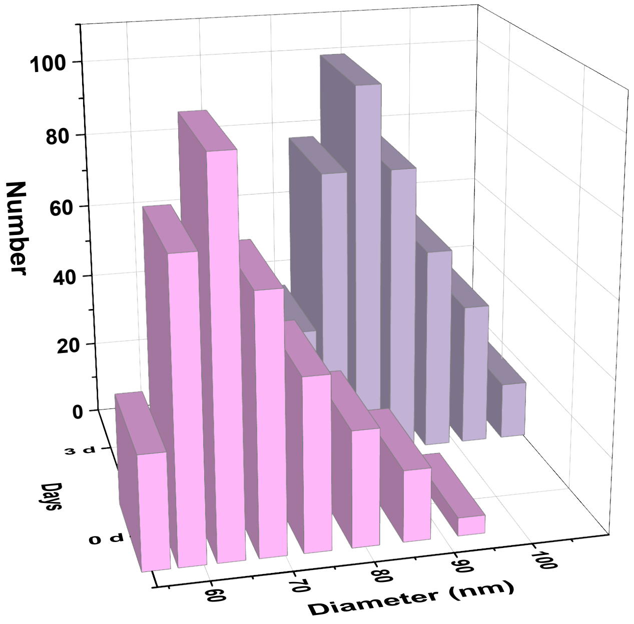

Figure S9. The diameter distribution of tsMIPs in PBS within 3 days.

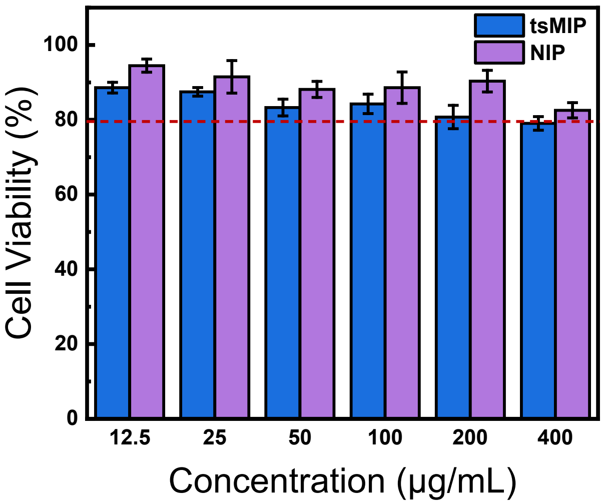

Figure S10. Cell viability analysis of tsMIP and NIP towards RAW264.7cells.

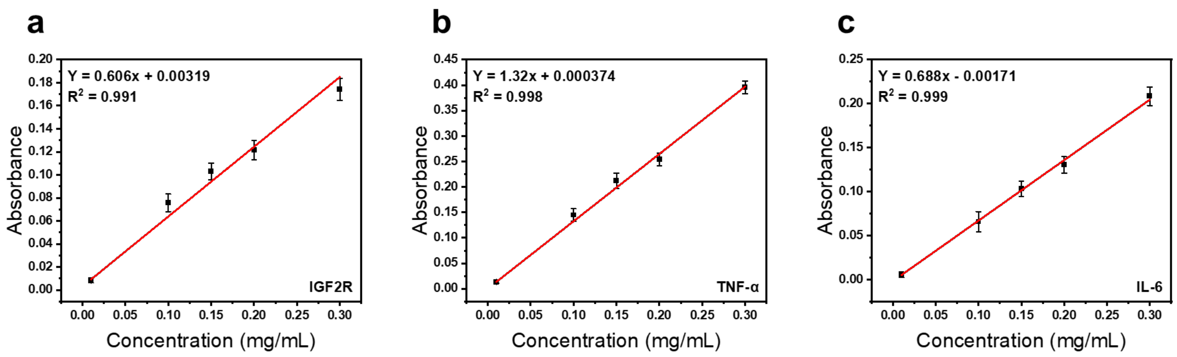

Figure S11. UV absorbance-based calibriation curves of the three epitopes. a) the N-terminal nonapeptide of IGF2R; b) the C-terminal nonapeptide of TNF-α; c) the C-terminal nonapeptide of IL-6.

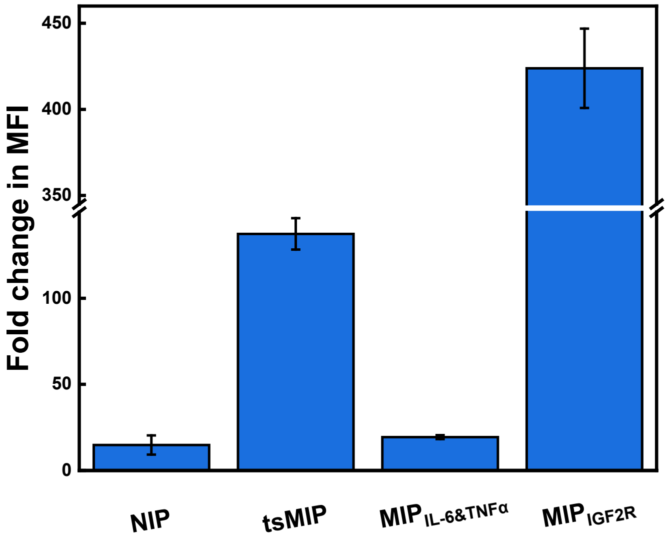

Figure S12. Fluorescence intensity analysis of flow cytometry experiment.

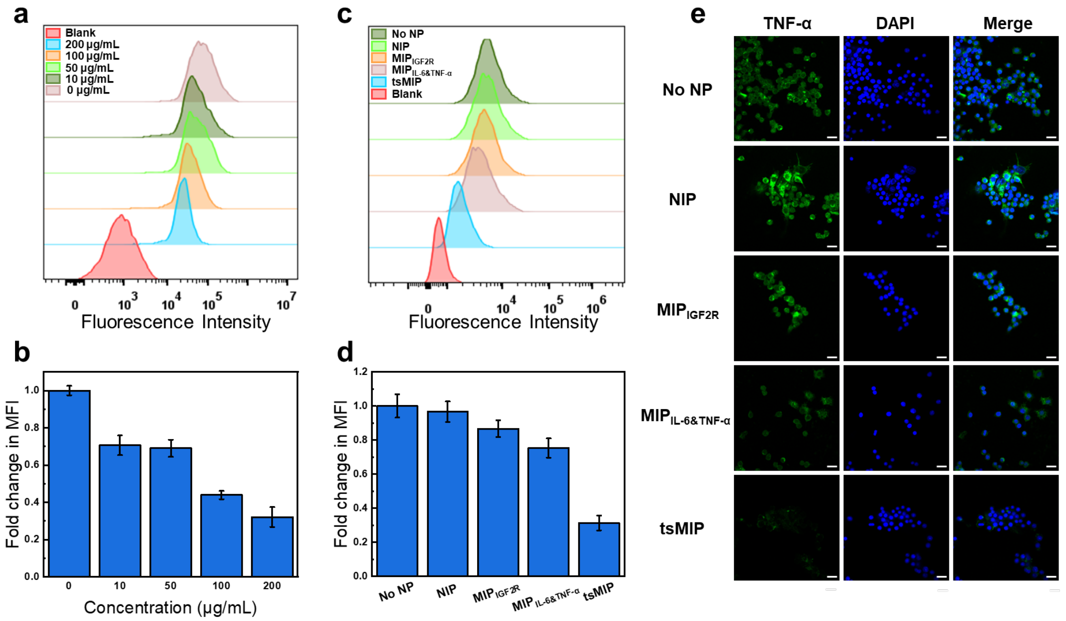

Figure S13. Investigation of tm-TNF-α degradation. a) Degradation of tmTNF-α on RAW264.7 cells measured by flow cytometry treated with tsMIP at different concentration; b) Fluorescence intensity analysis of flow cytometry experiments in (a); c) Degradation of tmTNF-α on RAW264.7 cells measured by flow cytometry treated with tsMIP or other control NPs; d) Fluorescence intensity analysis of flow cytometry experiments in (c); e) Visualization of cell surface TNF-α degradation on RAW264.7 cells by CLSM after the treatment with 100 μg/mL tsMIP or other NPs for 12 h. Scale bars = 20 μm. Error bars represent the standard deviations (n = 3).

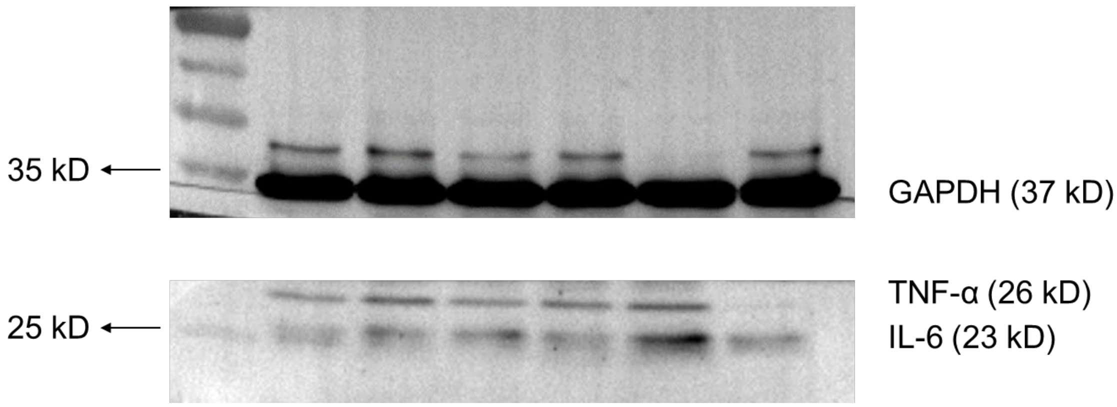

Figure S14. The origin image of Figure 5a.

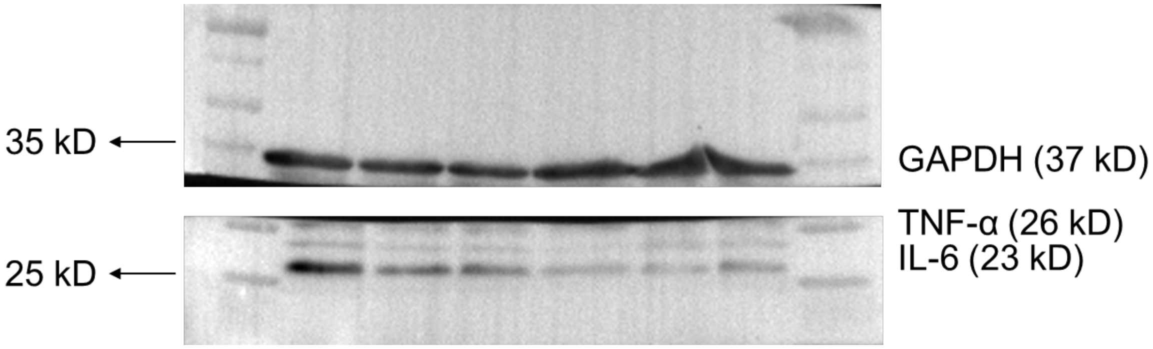

Figure S15. The origin image of Figure 5b.

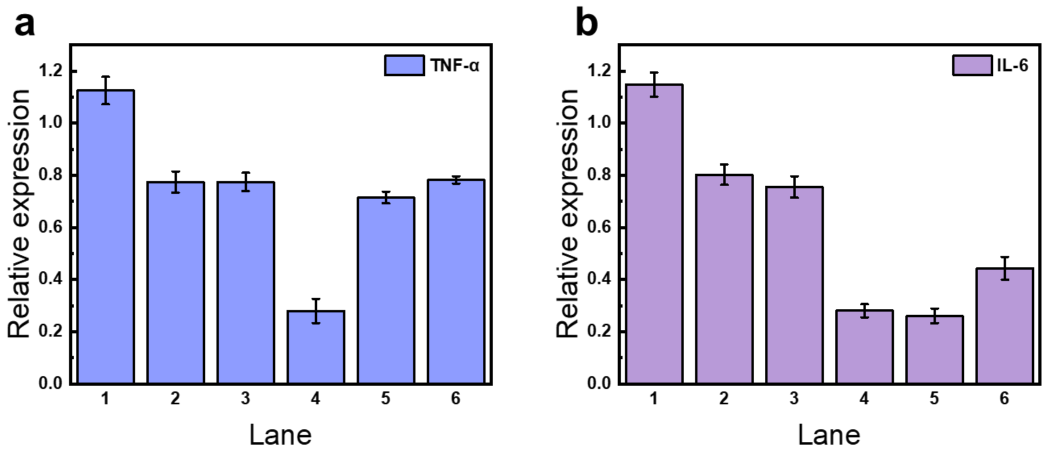

Figure S16. The quantitative analysis of Figure 5b. a) TNF-α; b) IL-6.

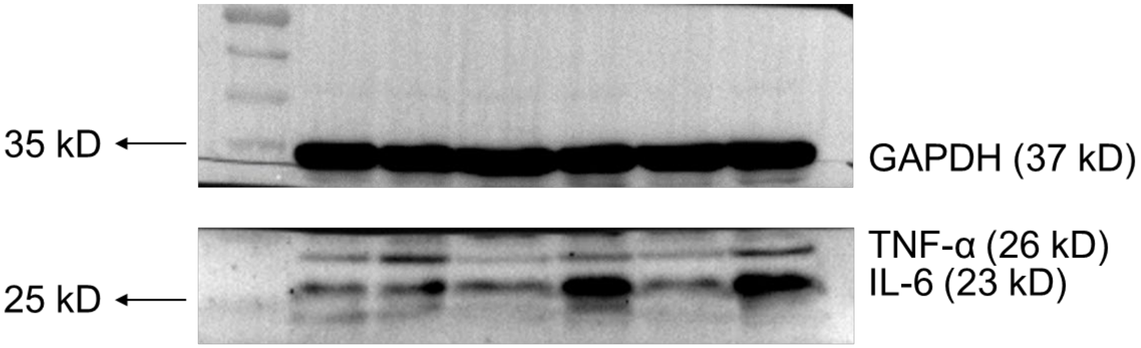

Figure S17. The origin image of Figure 5c.

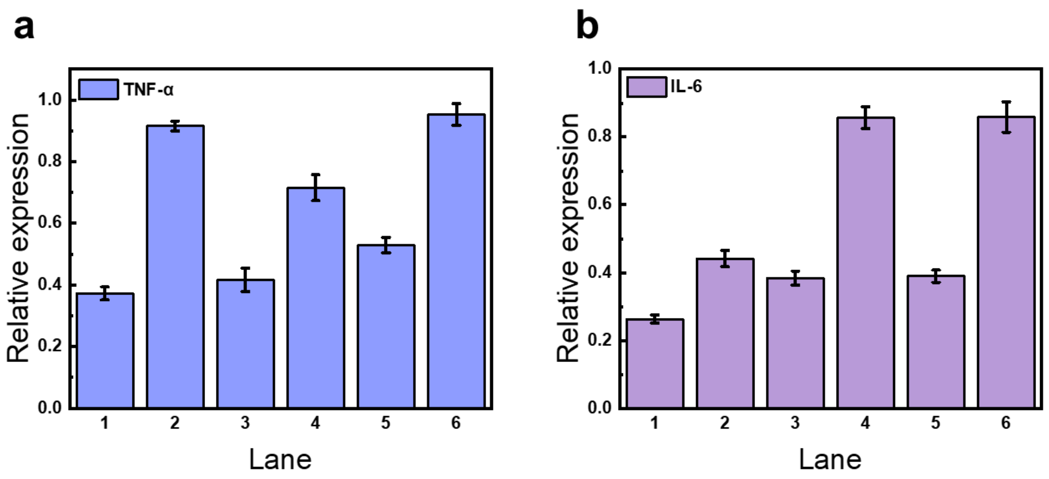

Figure S18. The quantitative analysis of Figure 5c. a) TNF-α; b) IL-6.

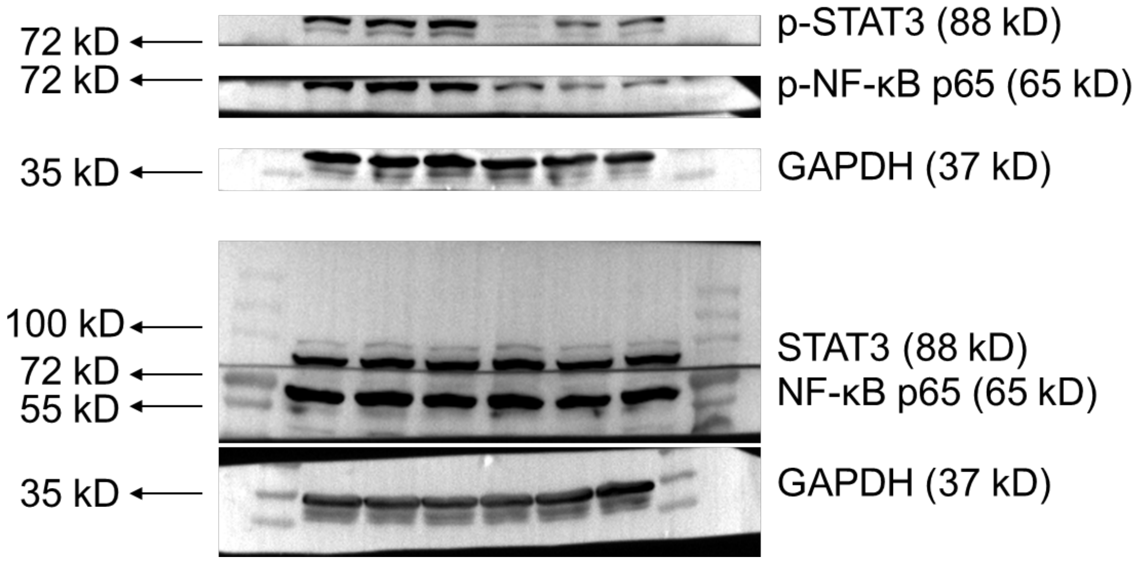

Figure S19. The origin image of Figure 5e.

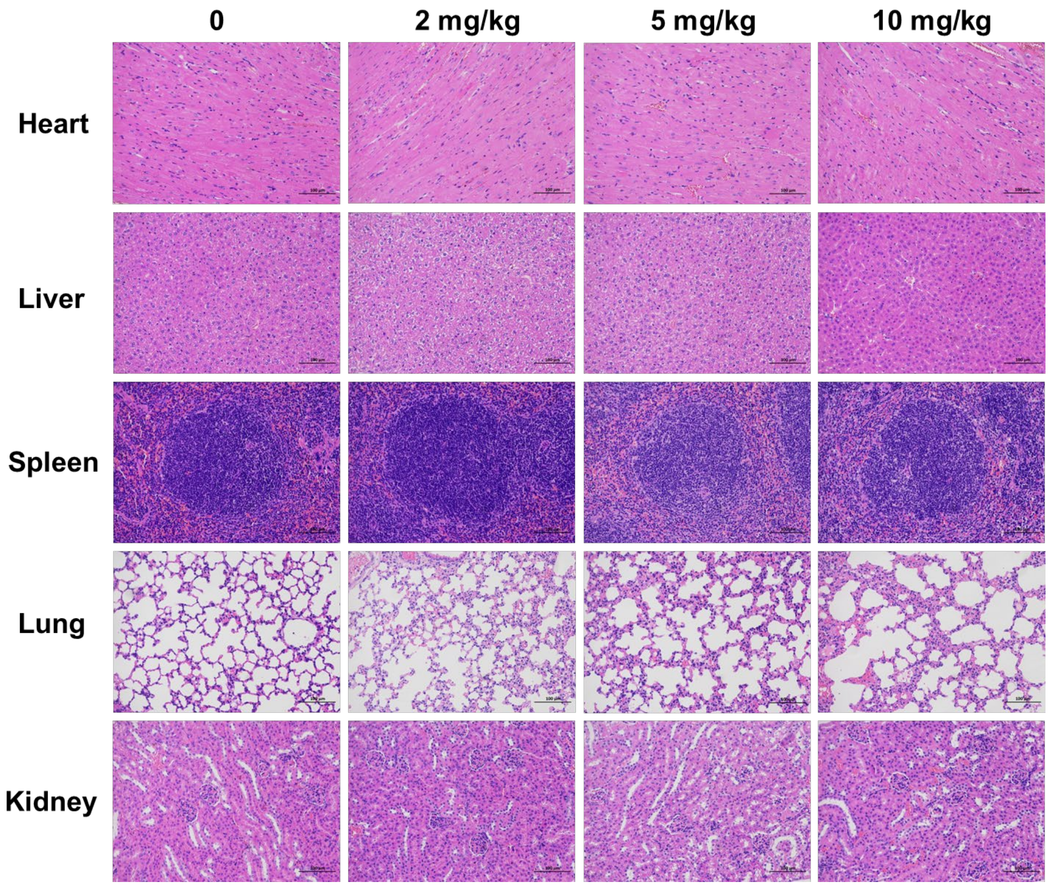

Figure S20. *In vivo* biosafety analysis of tsMIP. Hematoxylin-eosin (H&E) staining of organ tissues were obtained at 48 h of post different intranasal treatments.

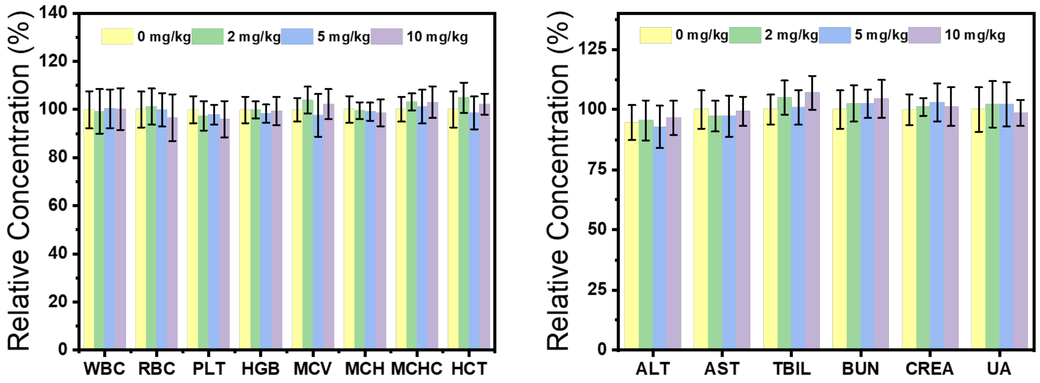

Figure S21. Biological toxicity study of different doses of tsMIP. (a) Blood analysis; (b) Hepatorenal function analysis.

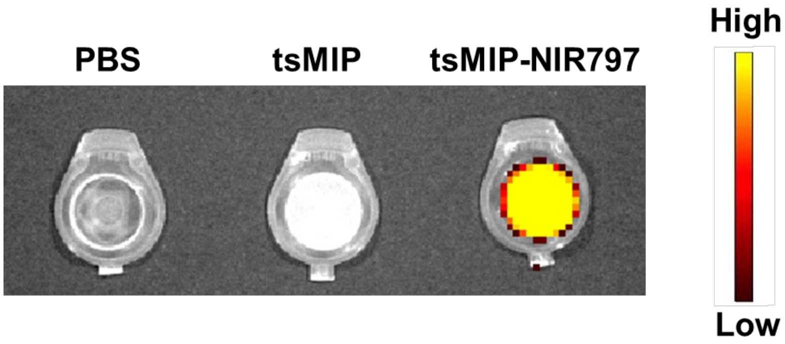

Figure S22. Fluorescence images of tsMIP, tsMIP-NIR797 dispersed in PBS solution and the bare PBS solution (1×).

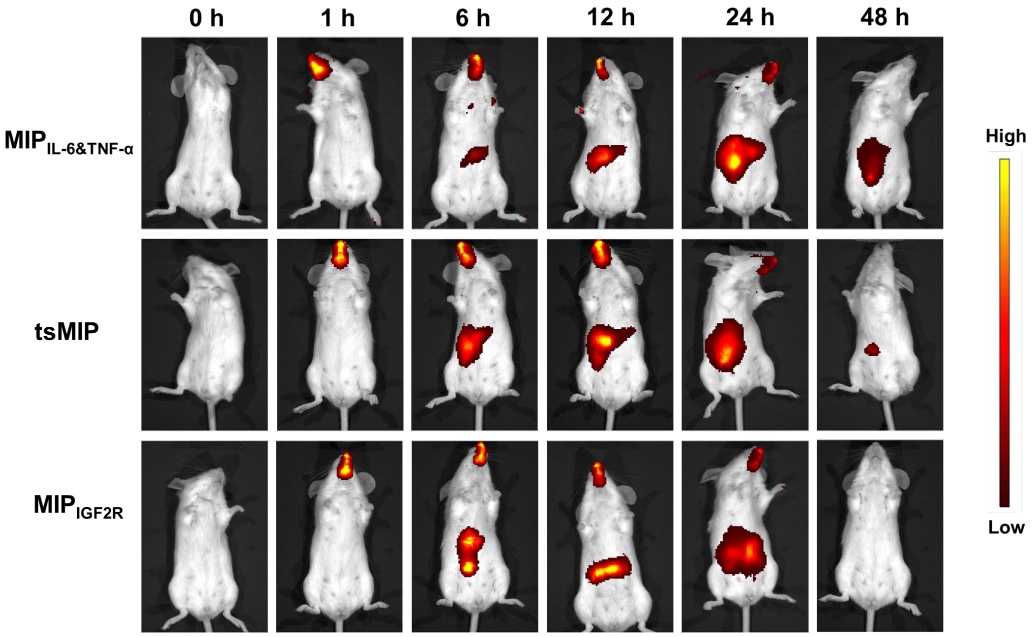

Figure S23. Biodistribution of tsMIP-NIR797, $MIP_{IL-6\&TNF-\alpha}$-NIR797 and MIP_{IGF2R}-NIR797 of different time intervals in BALB/c mice after being intranasally added. *In vivo* fluorescence images of intranasal tsMIP-NIR797 nanoparticles treated BALB/c mice at different time intervals.

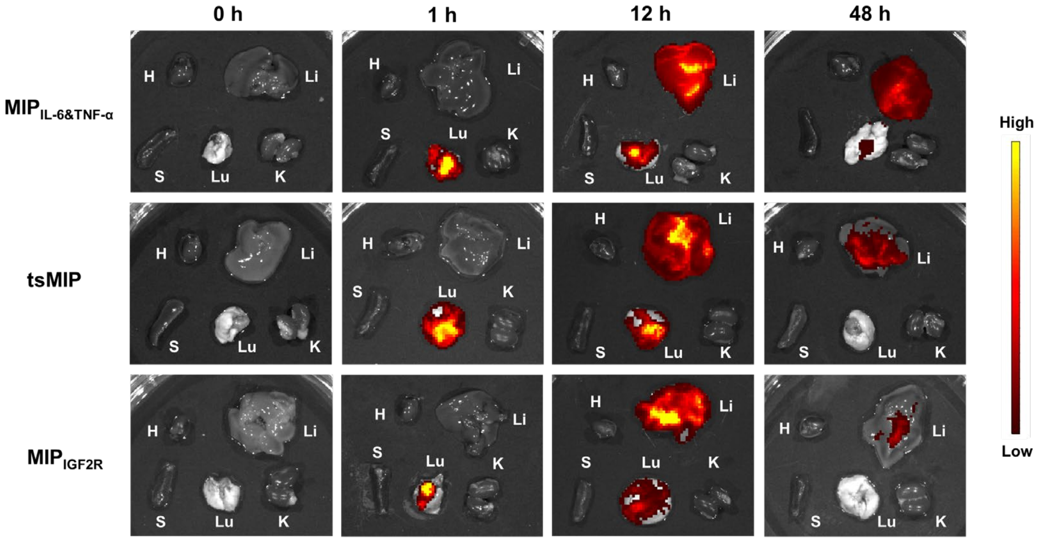

Figure S24. Biodistribution of tsMIP-NIR797, $MIP_{IL\text{-}6\&TNF\text{-}\alpha}$-NIR797 and MIP_{IGF2R}-NIR797 at different time intervals in BALB/c mice following intranasal administration. Fluorescence images of major organs dissected from intranasal tsMIP-NIR797, $MIP_{IL\text{-}6\&TNF\text{-}\alpha}$-NIR797 and MIP_{IGF2R}-NIR797 nanoparticles treated BALB/c mice at different time intervals. H: heart; Li: liver; S: spleen; Lu: lung; K: kidney.

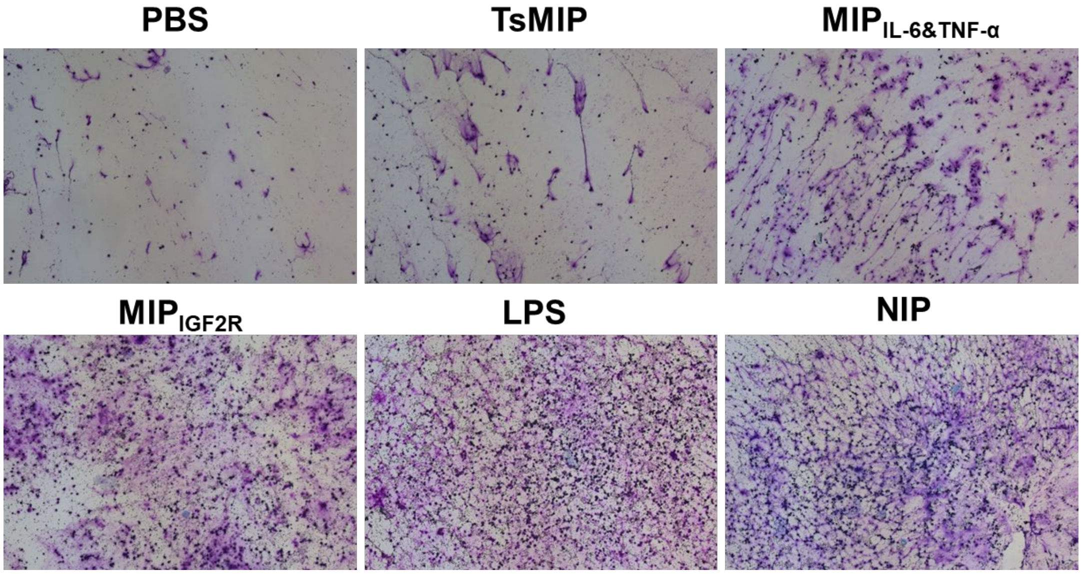

Figure S25. Analysis of inflammatory cells in BALF of different groups.

Supplementary References

1. J. Eberhardt, D. Santos-Martins, A. F. Tillack and S. Forli, *J. Chem. Inf. Model.*, 2021, **61**, 3891-3898.

2. Trott and A. J. Olson, *J. Comput. Chem.*, 2010, **31**, 455-461.

3. Z. Guo, R. Xing, M. Zhao, Y. Li, H. Lu and Z. Liu, *Adv. Sci.*, 2021, **8**, e2101713.

4. Y. Li, S. Xu, Q. Ye, H. Chi, Z. Guo, J. Chen, M. Wu, B. Fan, B. Li, C.-F. Qin and Z. Liu, *Advanced Science*, 2023, **10**, 2202689.
